# A Gene Transfer Agent and a Dynamic Repertoire of Secretion Systems Hold the Keys to the Explosive Radiation of the Emerging Pathogen *Bartonella*

**DOI:** 10.1371/journal.pgen.1003393

**Published:** 2013-03-28

**Authors:** Lionel Guy, Björn Nystedt, Christina Toft, Katarzyna Zaremba-Niedzwiedzka, Eva C. Berglund, Fredrik Granberg, Kristina Näslund, Ann-Sofie Eriksson, Siv G. E. Andersson

**Affiliations:** Department of Molecular Evolution, Cell and Molecular Biology, Science for Life Laboratory, Biomedical Centre, Uppsala University, Uppsala, Sweden; Universidad de Sevilla, Spain

## Abstract

Gene transfer agents (GTAs) randomly transfer short fragments of a bacterial genome. A novel putative GTA was recently discovered in the mouse-infecting bacterium *Bartonella grahamii*. Although GTAs are widespread in phylogenetically diverse bacteria, their role in evolution is largely unknown. Here, we present a comparative analysis of 16 *Bartonella* genomes ranging from 1.4 to 2.6 Mb in size, including six novel genomes from *Bartonella* isolated from a cow, two moose, two dogs, and a kangaroo. A phylogenetic tree inferred from 428 orthologous core genes indicates that the deadly human pathogen *B. bacilliformis* is related to the ruminant-adapted clade, rather than being the earliest diverging species in the genus as previously thought. A gene flux analysis identified 12 genes for a GTA and a phage-derived origin of replication as the most conserved innovations. These are located in a region of a few hundred kb that also contains 8 insertions of gene clusters for type III, IV, and V secretion systems, and genes for putatively secreted molecules such as cholera-like toxins. The phylogenies indicate a recent transfer of seven genes in the *virB* gene cluster for a type IV secretion system from a cat-adapted *B. henselae* to a dog-adapted *B. vinsonii* strain. We show that the *B. henselae* GTA is functional and can transfer genes *in vitro*. We suggest that the maintenance of the GTA is driven by selection to increase the likelihood of horizontal gene transfer and argue that this process is beneficial at the population level, by facilitating adaptive evolution of the host-adaptation systems and thereby expansion of the host range size. The process counters gene loss and forces all cells to contribute to the production of the GTA and the secreted molecules. The results advance our understanding of the role that GTAs play for the evolution of bacterial genomes.

## Author Summary

Viruses are selfish genetic elements that replicate and transfer their own DNA, often killing the host cell in the process. Unlike viruses, gene transfer agents (GTAs) transfer random pieces of the bacterial genome rather than their own DNA. GTAs are widespread in bacterial genomes, but it is not known whether they are beneficial to the bacterium. In this study, we have used the emerging pathogen *Bartonella* as our model to study the evolution of GTAs. We sequenced the genomes of six isolates of *Bartonella*, including two new strains isolated from wild moose in Sweden. Using a comparative genomics approach, we searched for innovations in the last common ancestor that could help explain the explosive radiation of the genus. Surprisingly, we found that a gene cluster for a GTA and a phage-derived origin of replication was the most conserved innovation, indicative of strong selective constraints. We argue that the reason for the remarkable stability of the GTA is that it provides a mechanism to duplicate and recombine genes for secretion systems. This leads to adaptability to a broad range of hosts.

## Introduction

Double-stranded DNA viruses are extremely abundant and evolve rapidly, yielding highly diverse viral populations. The transfer of bacteriophage DNA from one bacterial cell to another is regulated by the viral genome. Bacteriophage sequences may account for up to 20% of the bacterial chromosome, but most insertions are highly unstable and the presence/absence patterns of prophage genes vary even in otherwise nearly identical genomes. In generalized transduction, bacterial DNA is by mistake packaged into the phage capsid and transferred into another cell. Gene transfer agents (GTA) differ from viruses in that they transfer random pieces of the bacterial genome and that the fragments are shorter (<14 kb) than needed to encode the phage particle [1]. Although genes for putative gene transfer agents are widespread in bacterial genomes, the selective forces that drive their evolution and maintenance are still largely unexplored.

The best-studied agents are RcGTA from *Rhodobacter capsulatus*, which resembles a small, tailed bacteriophage and packages 4.5 kb DNA fragments [2], [3], [4], and VSH-1 in the intestinal spirochaete *Brachyspira* which transfers 7.5 kb DNA fragments [5], [6]. The 15 genes that encode the RcGTA are clustered, whereas the bacterial genes that control their expression are scattered around the *R. capsulatus* genome. Regulation is mediated by quorum-sensing systems and responds to changes in nutrition and stress in the environment [7]. Although functional RcGTA particles have so far only been identified in *Rhodobacter capsulatus* and *Ruegeria pomeroyi* [8] all members of the Rhodobacterales have complete RcGTA-like gene clusters, and most bacteria of other alphaproteobacterial orders contain partial clusters [4]. Phylogenies inferred from the capsid protein sequences show a similar topology as the 16S rDNA tree, indicating relationship by vertical descent [4]. The conservation of the RcGTA genes is remarkable given that lifestyles are extremely diverse and alphaproteobacterial genome sizes differ by one order of magnitude.

In this contribution, we have searched for factors that can explain the emergence of GTAs. We have used *Bartonella* as our model since they belong to the Alphaproteobacteria but have evolved their unique GTA [9] that is unrelated to the RcGTA [4]. Moreover, a peculiar amplification of a segment of several hundred kb in size has been observed in *Bartonella henselae* [10], [11] and *Bartonella grahamii* [9]. The peak of the amplification is located in a region that contains a few phage genes, suggesting that the origin of replication is derived from a bacteriophage [9], [11]. This resembles run-off replication (ROR) in *Salmonella*, where a phage origin in a prophage amplifies surrounding chromosomal sequences by accident [12]. Based on studies performed in *B. grahamii* it has been shown that the combination of the two phage-derived systems results in the production of phage particles that contain genomic DNA in direct proportion to the level of amplification from the ROR-region [9]. However, the evolutionary significance of the newly identified GTA in *Bartonella* has not yet been demonstrated.

*Bartonella* differ from most other members of the Rhizobiales and Rhodobacterales in that they are adapted to diverse mammalian hosts where they infect endothelial cells and erythrocytes. The infections are normally asymptomatic, but human pathogens like *Bartonella bacilliformis* and *Bartonella quintana* cause Oroya fever and trench fever, respectively. Because of the many hosts involved, and the opportunity for transmission to novel hosts with the aid of blood-sucking arthropods, *Bartonella* is also a good model organism for studies of the molecular mechanisms involved in adaptive radiation [13].

The acquisition of a type IV secretion system (VirB) and the associated genes for effector proteins have been suggested to represent the key innovation event that triggered adaptive radiation in two *Bartonella* lineages [13]. The *virB* operon encodes a pilus structure that injects a combination of effector proteins directly into the primary host cell niche, causing modulations of a variety of host cytoplasmic functions [14], [15], [16]. It was hypothesized that the *virB* gene cluster was transferred from a conjugative plasmid into the ancestral strains of these two lineages in two separate events [13]. Another gene cluster for a conjugative T4SS, *trw*, which mediates binding to erythrocytes [17], has also been imported into *Bartonella* from a plasmid [18]. Both the *virB* and the *trw* gene clusters are necessary for successful infections of *B. tribocorum* in a rat model [17], [19]. Although it is generally agreed that the surface components of these systems evolve at high rates, different mechanisms have been proposed. Positive selection for nucleotide substitutions is one hypothesis [13], higher fixation rates for recombination events due to diversifying selection is another [10], [18].

Despite remarkable progress in our understanding of the function of the different T4SSs in *Bartonella*, a comprehensive understanding of the evolution and plasticity of their genomes is still lacking. Here, we present the sequences of six new genomes of non-pathogenic isolates from wild and domestic animals and connect the discovery of the unique GTA with the acquisition and evolution of several different host adaptation systems. We propose that the acquisition of secretion systems along with a phage-derived system to modify them underlies adaptive radiation of all lineages in the genus *Bartonella*.

## Results/Discussion

### *Bartonella* genome features and statistics

Whole genome shotgun sequencing was performed on six *Bartonella* isolates (Table 1). The isolates were selected to provide a broad sampling of the known phylogenetic diversity of the genus *Bartonella*. Four isolates have been described previously, including strains from a marsupial, *B. australis* NH1 [20], a cow, *B. bovis* (Bermond) 91-4 [21] and two dogs, *B. vinsonii berkhoffii* Winnie isolated from a Pekingese [22] and Tweed isolated from a Labrador [23]. Two isolates were obtained as part of this study; both were cultivated from blood samples taken from moose at two different sites in Sweden. These isolates were most similar to *B. bovis* and *B. schoenbuchensis* based on 16S rRNA sequence data, and were classified as *B. bovis* m02 and *B. schoenbuchensis* m07a, respectively. Experimental infection of bovine endothelial cells by the moose isolate *B. bovis* m02 is shown in Figure S1.

**Table 1:** *Bartonella* isolates.

Organism	ID	Host	Country	Acc. No	Lineage	Group	Ref.
*B. australis* NH1^T^	BAnh1	Kangaroo (*Macropus giganteus*)	Australia	CP003123			[20]
*B. bacilliformis* KC583^T^^,^ P	BB	Human (*Homo sapiens*)	Peru	NC_008783	1		[90]
*B. bovis* (Bermond) 91-4^T^	BBb	Cattle (*Bos taurus*)	France	AGWA00000000	2	A	[21]
*B. bovis* m02	m02	Moose (*Alces alces*)	Sweden	AGWB00000000	2	A	
*B. schoenbuchensis* m07a	m07a	Moose (*Alces alces*)	Sweden	AGWC00000000	2	A	
*B. schoenbuchensis* R1	BSc	Roe deer (*Capreolus capreolus*)	Germany	FN645506-24	2	A	[13]
*B. rochalimae* ATCC BAA-1498	BRo	Dog (*Canis lupus*)/various canids	USA	FN645455-67	3	B	[13]
*B.* sp. 1-1C	B11	Rat (*Rattus norvegicus*)	Taiwan	FN645486-505	3	B	[13]
*B.* sp. AR 15-3	BAR	Squirrel (*Tamiasciurus hudsonicus*)	USA	FN645468-85	3	B	[13]
*B. clarridgeiae*	BC	Cat (*Cattus felis*)	France	NC_014932	3	B	[13]
*B. quintana* Toulouse^P^	BQ	Human (*Homo sapiens*)	France	NC_005955	4	C	[30]
*B. henselae* Houston-1^T^^,^ P	BHH1	Human (*Homo sapiens*)*	U.S.A.	NC_005956	4	C	[30]
*B. vinsonii berkhoffii* Winnie	BVwin	Dog [Pekingese] (*Canis lupus*)	U.S.A.	CP003124	4	C	[22]
*B. vinsonii berkhoffii* Tweed	BVtw	Dog [Labrador] (*Canis lupus*)	U.S.A.	AGWD00000000	4	C	[23]
*B. grahamii* as4aup	BG	Wood mouse (*Apodemus sylvaticus*)	Sweden	NC_012846-7	4	C	[9]
*B. tribocorum* IBS 325^T^	BT	Rat (*Rattus norvegicus*)	France	NC_010160-1	4	C	[24]

T Type strain.

P Human pathogen.

* Cats are the supposed natural reservoir of *B. henselae.*

The six new genomes are at various stages of completion, including two fully resolved genomes of *B. australis* NH1 and *B. vinsonii* Winnie (Table S1). Unresolved gaps in the draft genomes covered phage genes in *B. vinsonii* Tweed and long stretches of approximatively 20–100 kb of repeated genes with internal repetitions in *B. bovis* m02 and 91-4, and *B. schoenbuchensis* m07a (Figure S2). The architectures of the *Bartonella* genomes, as inferred from comparisons to previously sequenced genomes are largely conserved, with the exception of a major inversion in *B. bacilliformis*, which was confirmed by PCR and sequencing, and several smaller inversions across the terminus of replication (Figure S2). All single-copy genes and at least one copy of all duplicated genes were resolved in all draft genomes. For consistency, the newly sequenced genomes were annotated along with the previously published genomes through our pipeline using the manually annotated *B. grahamii* genome as the reference (Figure S3).

Plasmids of 23–28 kb have previously been identified in *B. grahamii* (pBGR3, NC_012847) [9] and *B. tribocorum* (plasmidBtr, NC_010160) [24], both of which include a copy of *vbh*, a type IV secretion system (T4SS). Contigs 8 to 10 in *B. schoenbuchensis* R1 (FN645513-15) are also likely derived from a plasmid, which we have here designated pSc. The m07a strain of *B. schoenbuchensis* sequenced as part of this study harbors two plasmids, one of which is 59 kb (pML) and shows homology to pBGR3 and pSC. The other (pMS) is only 2 kb and contains only three genes (two copies of *repA*, *mob*) that shows homology to genes on the 2.7 kb cryptic plasmids pBGR1 and pBGR2 identified in *B. grahamii* isolate IBS 376 and WM10, respectively (NC_006374, NC_004308) [25].

### The *Bartonella* species phylogeny

Combined with previously published data, a total of 16 *Bartonella* genomes (8 complete and 8 draft genomes) were included in our comparative genomics study (Table S2). These genomes range in size from 1.4–2.6 Mb, comprising about 1100–2000 CDSs per genome. To place the genomic data in an evolutionary context, we inferred a reference phylogeny of the 16 *Bartonella* isolates and 6 representative outgroup species that are also members of the Rhizobiales. For this purpose, we clustered the encoded proteins into families of protein homologs using a Markov chain algorithm [26]. Core genes present in a single copy in all genomes were extracted from the clustered families and complemented with ribosomal protein genes (which are duplicated in *B. bacilliformis*) and *groEL*, *groES* and *gltA* (which are duplicated in the outgroup species). This resulted in a dataset of 428 genes. The mean substitution frequencies for these genes was estimated to less than 0.12 substitutions per nonsynonymous site (Ka) and up 1.09 substitutions per synonymous site (Ks) in all pairwise comparisons within the genus *Bartonella* (Table S3). Synonymous substitution frequencies between *Bartonella* species and the outgroup were above saturation level (Table S3).

**Figure 1:**
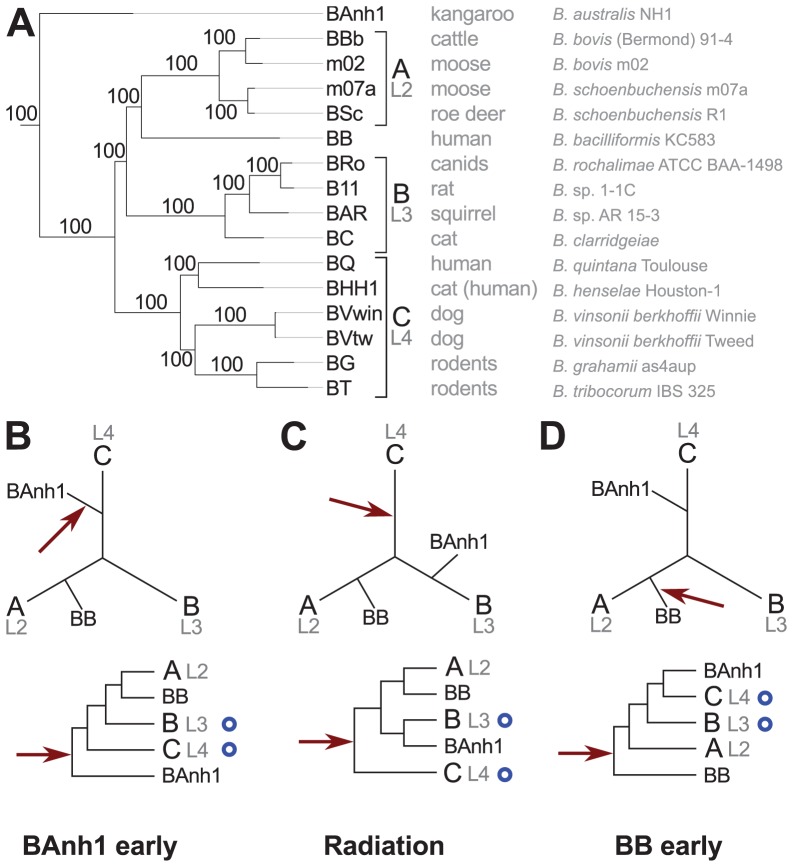
Phylogenetic relationships of the *Bartonella* species examined in this study. Phylogenetic tree inferred from a maximum likelihood analysis of a concatenated alignment of 428 genes (A) and three schematic figures showing different placements of *B. australis* (BAnh1) and the root (B–D). The most common host of each strain and the full name of the strain are indicated next to the tree in (A). Numbers on the branches indicate bootstrap support in the maximum likelihood phylogeny. Lineages (L2-4) refer to the nomenclature used in [13]. The schematic phylogeny depicted in (B) is taken from the phylogenetic analyses presented in Figure S5A, which suggest that *B. australis* diverges early (“BAnh1 early”). (C) The schematic phylogeny depicted in (B) is taken from the phylogenetic analyses presented in Figure S5C, which suggests that *B. australis* is a sister clade to group B (“Radiation”). The schematic phylogeny depicted in (D) is taken from the phylogenetic analyses presented in [13], which suggest that *B. bacilliformis* (BB) diverges early (“BB early”). The position of the root is depicted with a red arrow (B–D). Blue circles indicate the presence of the VirB type IV secretion system.

The phylogenetic analyses based on the concatenated nucleotide alignment using maximum likelihood and Bayesian methods revealed three highly supported groups (Figure 1A). Group A includes the ruminant-adapted strains, group B contains isolates from cats, rats and squirrels and group C is composed of the human pathogen *B. quintana* and of strains isolated from cats, dogs and rodents. Moreover, the phylogeny placed *B. australis* as the earliest diverging species with high support (bootstrap support = 100%; posterior probability = 1.0). This finding was surprising since earlier studies have placed *B. australis* near to the C-group strains *B. grahamii* and *B. tribocorum* [20]. We tested five possible placements of *B. australis* in single gene trees using both nucleotide and amino acid alignments, and found that in 67% to 85% of all trees the likelihood was the highest for a placement as either the earliest diverging species (Figure 1B, same topology as inferred from the concatenated nucleotide alignment, Figure 1A) or as a sister species to group B (Figure 1C) (Figure S4). Only 5% to 15% of the trees indicated a placement near to group C.

The *B. australis* genome is slightly more GC-rich than the other genomes and we reasoned that its placement as the deepest diverging species could be due to attraction to the more GC-rich outgroup taxa for rapidly evolving genes. Indeed, sequence divergence level was the factor that most strongly correlated with tree topologies: the most divergent gene sets placed *B. australis* as the earliest diverging species (pp = 0.98; bootstrap = 77–93%) (Figure 1B), whereas the least divergent gene sets placed *B. australis* as a sister taxa to the B-group strains (pp = 0.94; bootstrap = 90–95%) (Figure 1C) (Figure S5). Consistently, a t-test revealed a significant (p = 0.039) correlation between the mean Ka-values and the placement of *B. australis* as the deepest diverging lineage. To examine the support for the rooting, we calculated the likelihood of seven alternative placements of the root. In 70% of the single gene trees inferred from nucleotide sequences but in only 35% of the trees built from amino acid alignments, the highest likelihood was associated with a rooting on the *B. australis* branch (Figure 1B) (Figure S6).

To further improve the rooting in the analysis, we included *Bartonella tamiae* Th239, whose draft genome was recently released in Genbank. This was for two reasons: the genomic GC content is 38%, which is similar to the GC content of the other *Bartonella* genomes, and a previous phylogenetic analysis based on the neighbor-joining method with the *gltA* gene indicated that it is a very early diverging species, although there was no bootstrap support for this placement [27]. To reduce the influence of putative horizontal gene transfers, we applied a discordance filter to a concatenated amino-acid alignment of 425 proteins [28] in which 0 to 40% of the proteins producing the most deviant tree topologies were removed. The tree topology obtained from an alignment where 10% of the genes were removed showed that *B. tamiae* is the earliest diverging species with 100% bootstrap support, followed by *B. australis*, with 96% bootstrap support (Figure S7). Importantly, the topology was unchanged even after the removal of up to 40% of the most discordant genes (Figure S8). This suggests that the tree topology is not affected by the inadvertent inclusion of a subset of horizontally transferred genes with a different evolutionary history. Moreover, the analysis provided additional support to the hypothesis for the placement of *B. australis* as the earliest diverging species (excluding *B. tamiae*), although a small set of highly conserved genes indicates that it clusters with the B group strains.

Both of these two topologies (Figure 1B and 1C) are in conflict with the proposal that *B. australis* is related to the rodent strains *B. grahamii* and *B. tribocorum* [20]. Since we have sequenced the exact same strain as deposited by the authors [20], the discrepancy resides in the use of different genes and gene sequences for the phylogeny. We argue that the previous clustering with rodent *Bartonella* species was a PCR artifact since only 4 of the 6 *B. australis* genes that were used to construct the published tree are identical in sequence to those of the *B. australis* genome, and none of these 4 genes, including the *gltA* gene, supported a grouping with the rodent isolates (data not shown). Consistently, a previously published phylogeny of *Bartonella* species based on the *gltA* gene also indicated that *B. australis* clusters separately from *B. grahamii* and *B. tribocorum* [27]. The other two PCR amplified genes (*rpoB* and *ftsY*) differ in sequence from those of the *B. australis* genome, suggesting that they may have been amplified from DNA of a contaminating rodent *Bartonella* species. This would explain the affiliation of *B. australis* with *B. grahamii* and *B. tribocorum* in the study by Fournier et al. [20].

**Figure 2:**
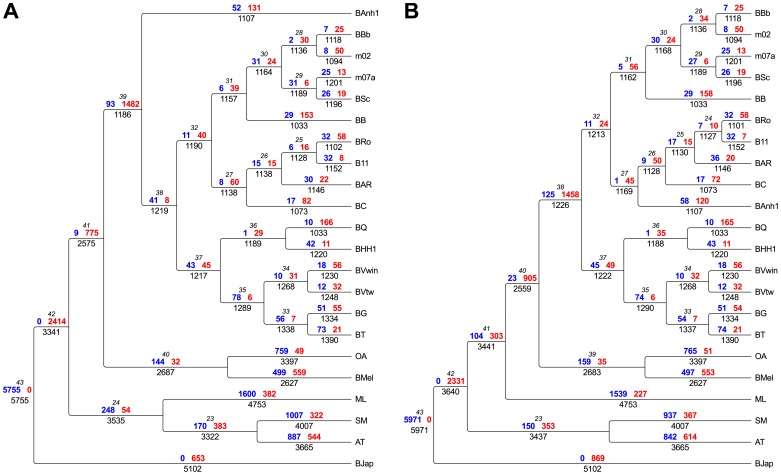
Flux of protein families in *Bartonella* and related outgroup species. The flux of protein families has been mapped onto the two most probable phylogenies, “BAnh1 early” shown in Figure 1B) (A) and “Radiation” shown in Figure 1C (B). Genesis (blue) and loss (red) of protein families are depicted above each branch, respectively. Total number of gene families is indicated below the branch. The node number is shown in italics. Abbreviations of *Bartonella* species are as in Table 1. Abbreviations of outgroup species: AT, *Agrobacterium tumefaciens;* SM, *Sinorhizobium meliloti*; ML, *Mesorhizobium loti*; BMel, *Brucella melitensis*; OA, *Ochrobactrum anthropi*; BJap, *Bradyrhizobium japonicum*.

The clustering of *B. bacilliformis* with the ruminant A-group strains was consistently observed in all analyses (Figure 2; Figures S4, S5, S6, S7, S8). This finding is notable since all previous attempts to identify a sister clade of *B. bacilliformis* that could provide indications of its natural host reservoir have failed. The lack of affiliation with other *Bartonella* species has been taken as an argument to suggest that *B. bacilliformis* represents the earliest diverging species in the genus and that this species has retained more ancestral features than the other species [13], [24], [29] (Figure 1D). Our phylogeny is inconsistent with this hypothesis, and, to the best of our knowledge, no other circumstantial data suggest that *B. bacilliformis* is more “ancestral” than the other species. The new placement of *B. bacilliformis* as a sister clade to Group A is most likely due to the inclusion of *B. australis* and as many as four different A-group species in our concatenated genome tree.

Rather than representing ancestral features, the small genome size and the high virulence properties of *B. bacilliformis* are characteristic of reductive genome evolution following a recent host switch, as observed for *B. quintana*, the agent of trench fever [30] and *Rickettsia prowazekii*, the agent of epidemic typhus [31]. In analogy, we suggest that the establishment of *B. bacilliformis* in the human population was a rare event that was started from a small founder population that successfully made a host shift from ruminants, possibly from local camelids, to humans. The global distribution of ruminant *Bartonella* species and ruminant-infecting vectors represent a large reservoir of ruminant-infecting *Bartonella* species. But even if incidental infections by ruminant *Bartonella* species occur frequently, the lack of global vector systems may restrict their establishment in the human population. Indeed, the small geographic area in South America in which infections with *B. bacilliformis* occurs is thought to correspond to the geographic area of the sandfly transmitting the pathogen [32].

### Building the foundation for the explosive radiation

Having inferred a robust species phylogeny, we set out to identify genes acquired in the *Bartonella* last common ancestor (BLCA), thereby providing clues to the basis of the subsequent radiation of *Bartonella* species with different host preferences. To this end, we clustered all CDSs in the 16 *Bartonella* genomes and the outgroup species into families of protein homologs, resulting in 11,315 protein families of which 1828 families contained *Bartonella* proteins. Some families contain only orthologs encoded by single-copy genes whereas other families are larger and contain several paralogous proteins. We inferred the loss and gain of the protein families along the branches of the tree (Figure 2) with parsimony character mapping [33], [34]. For the interpretation of the gene content analyses we have considered both of the supported topologies (“BAnh1 early”, Figure 1B, and “Radiation”, Figure 1C). The analysis revealed in both cases the loss of about 1,500 protein families in the BLCA, which was balanced by the acquisition of about 100 protein families (Figure 2), resulting in a large efflux of genes. This estimate of acquired functions does not include duplicated and rapidly evolving surface proteins (see below) that are too divergent in sequence and/or size to be included in distinct protein families, given the criteria used for protein clustering.

#### A novel gene transfer agent is the most conserved innovation

**Figure 3:**
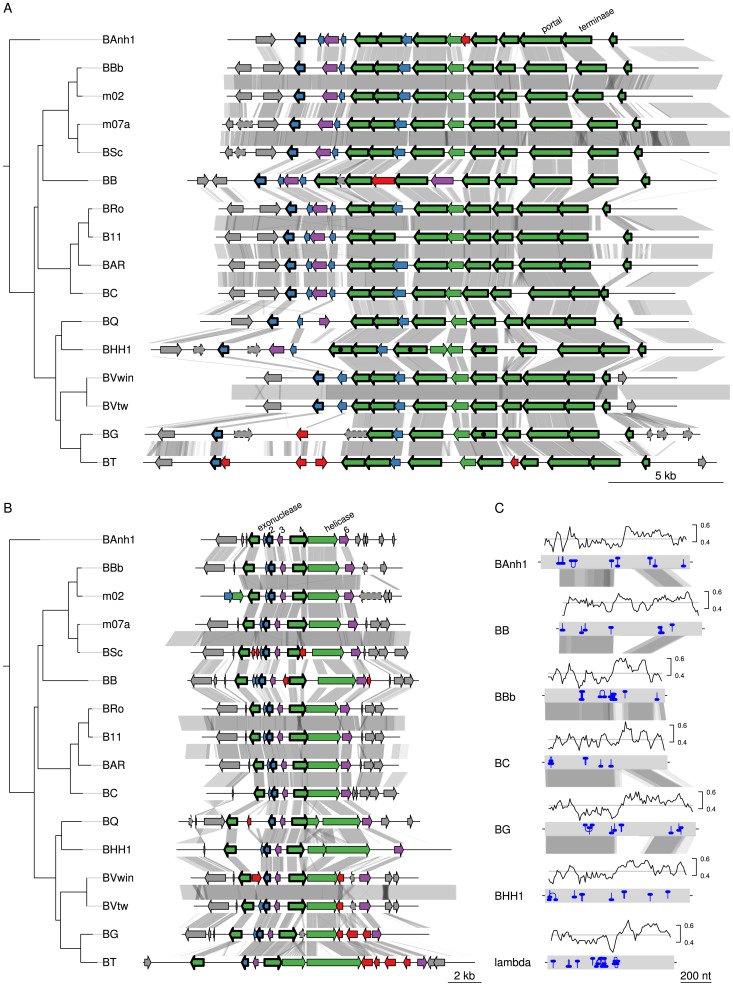
Gene order structures of the BaGTA and ROR segments. Comparative analysis of the DNA segments covering the BaGTA genes (A) and the ROR genes (B). Genes are colored according to their phylogenetic classification (see methods), without manual intervention: vertically inherited (magenta), imported (green), *Bartonella*-specific (blue) and ORFans (red). Genes acquired in the common ancestor of *Bartonella* and conserved in length and present in all genomes are marked with a thick border around the arrow depicting the gene. Those that are not bordered were presumably acquired in the common ancestor but have since then been lost or slightly modified in one or a few species. Pseudogenes are marked with a dotted border. Proteins experimentally detected by mass-spectrometry as part of the BaGTA phage particles [9] are marked with a filled black circle inside the arrow depicting the gene in *B. henselae* or *B. grahamii*. Genes shown in grey represent core genes not part of the BaGTA or the ROR. Horizontal grey lines indicate tblastx hits, with grey intensity reflecting the tblastx e-values. The repeat structure and GC content of gene number 4 in the ROR region is shown in (C). Blue lines indicate palindromic repeats. The graphs above the gene (grey bands) show the GC content in a 50-bp window. The average is indicated with a horizontal line. Abbreviations of *Bartonella* species names are as in Table 1.

We examined in greater detail the 93 novel acquisitions in the BLCA, suggested by the gene flux analysis presented in Figure 2A. Phage-related functions represented the largest category of acquired genes with predicted functions encompassing 23 protein families, while as many as 48 protein families represent hypothetical proteins of unknown functions (Table S4). Of the 93 putatively novel protein families, 22 families (covering 25 genes in *B. australis*; Table 2) are conserved in size and sequence among all genomes analyzed in this study. Genes for twelve of these 22 families are organized into two gene clusters that encode phage functions; a longer segment of 20 to 25 kb contains 9 genes and a shorter segment covers 3 genes. It has previously been suggested that the 9 co-located genes encode a *Bartonella* gene transfer agent (BaGTA) in *B. grahamii* [9]. Consistently, our study showed that 4 of the 9 co-located genes are homologous to typical phage proteins such as capsid, portal and terminase proteins (E-value<e-30) (Table 2; Figure 3A).

**Table 2:** Genes of *B. australis* belonging to the 22 protein families^a^ that are novel in *Bartonella* and retained in all genomes examined here.

Start	End	Cluster	Length	Gene	Locus tag^b^	Product
200471	201322	-	852	-	BAnh101740	cell wall hydrolase SleB
261494	262090	-	597	exo	BAnh102240	phage-related exonuclease
399858	401213	-	1356	-	BAnh103270	sodium/dicarboxylate symporter
407515	409218	-	1704	-	BAnh103340	hypothetical protein
480201	480968	-	768	cycH	BAnh103910	cytochrome c-type biogenesis protein CycH
611537	612700	-	1164	-	BAnh104870	putative virulence determinant
673279	673557	-	279	-	BAnh105340	hypothetical protein
753310	753801	-	492	-	BAnh106010	hypothetical protein
992500	993066	-	567	-	BAnh107950	hypothetical protein
1073527	1074066	-	540	-	BAnh108550	hypothetical DNA-binding protein
1194931	1195851	-	921	-	BAnh109520	putative phage tail fiber protein
1222495	1223091	-	597	exo	BAnh109840	phage-related exonuclease
1368045	1368491	BaGTA	447	-	**BAnh110810**	hypothetical membrane protein
1370722	1371693	BaGTA	972	-	**BAnh110850**	putative phage tail fiber protein
1371690	1372802	BaGTA	1113	-	**BAnh110860**	hypothetical membrane protein
1373314	1374777	BaGTA	1464	-	**BAnh110880**	hypothetical protein
1375918	1377036	BaGTA	1119	-	**BAnh110910**	phage related protein
1377179	1378018	BaGTA	840	-	**BAnh110920**	hypothetical protein
1378067	1379929	BaGTA	1863	-	**BAnh110930**	putative phage portal protein
1379929	1381272	BaGTA	1344	-	**BAnh110940**	phage terminase, large subunit
1382071	1382400	BaGTA	330	-	**BAnh110950**	hypothetical protein
1395225	1395860	ROR	636	exo	**BAnh111070**	phage-related exonuclease
1396315	1396704	ROR	390	-	**BAnh111090**	hypothetical protein
1397835	1398893	ROR	1059	-	**BAnh111110**	phage related protein
1428420	1429094	-	675	thiE2	BAnh111430	thiamine-phosphate pyrophosphorylase ThiE

a With protein families, we mean families of homologous proteins as identified during the clustering of all CDSs in the 16 *Bartonella* genomes and the outgroup species. Some families contain proteins encoded by single-copy genes whereas other families are larger and contain several paralogous proteins.

b Locus tags for genes located in clusters in BaGTA or ROR are marked in bold.

The other three genes are part of a six-gene conserved phage remnant, encoding among other functions a phage exonuclease and a phage helicase and containing a phage-derived origin of replication (Table 2; Figure 3B). By analogy to run-off replication in which replication of chromosomal DNA is initiated from a phage-derived origin of replication in *E. coli*, we have termed this region ROR (Run-off replication). The order and orientation of genes in this region is compatible with a lambda-like initiator-helicase loader replication module where the origin of replication is located in the O-protein-encoding gene upstream of the helicase gene [35]. The conserved gene 4, which is located upstream of the helicase gene in all *Bartonella* genomes, shares features with the lambda O-protein gene: both have a dip in GC content close to the O-protein binding site, followed by a peak in GC content. Moreover, the O-protein binding site is composed of four (relaxed) inverted repeats, and similar palindromic structures can be observed inside gene 4 (Figure 3C).

To investigate the rate of sequence evolution of the BaGTA, we calculated nonsynonymous and synonymous substitution frequencies (Ka and Ks, respectively) in pairwise comparisons within each lineage, averaged over the whole tree. We estimated the Ka and Ks values to respectively 0.098 and 0.54 substitutions per site for the genes putatively encoding the BaGTA (Table S5), as expected for genes that evolve under purifying selection. We compared these estimates to those of the flanking core genes, here defined as collinear, orthologous genes of similar sizes present in all genomes of a lineage. The analyses showed similar substitution frequencies for the genes coding for the BaGTA as for the neighboring core genes (Table S5): neither Ka nor Ks values were significantly different (Welch two-sample t-tests, p-values>0.3). Similarly, the genes in the ROR region showed similar Ka and Ks values as the core genes (Table S5). Again, neither Ka nor Ks were significantly different from the neighboring core genes (Welch two-sample t-tests, p-values>0.25). All these estimates are well within the range of Ka values (average = 0.044 substitutions per site) and Ks values (average = 0.37 substitutions per site) for all core genes in the genome overall (Table S5). The identification of phage genes in *Bartonella* that are conserved in both sequence and gene orders is remarkable since most other phage genes are highly dynamic and differ in gene presence/absence pattern even among *Bartonella* strains that are otherwise almost identical in sequence [11]. Taken together, this suggests that the gene clusters for BaGTA and the phage-derived origin of replication evolve under strong selective constraints, similar to other core genes.

#### The gene transfer agent is located near a secretion system cassette

**Figure 4:**
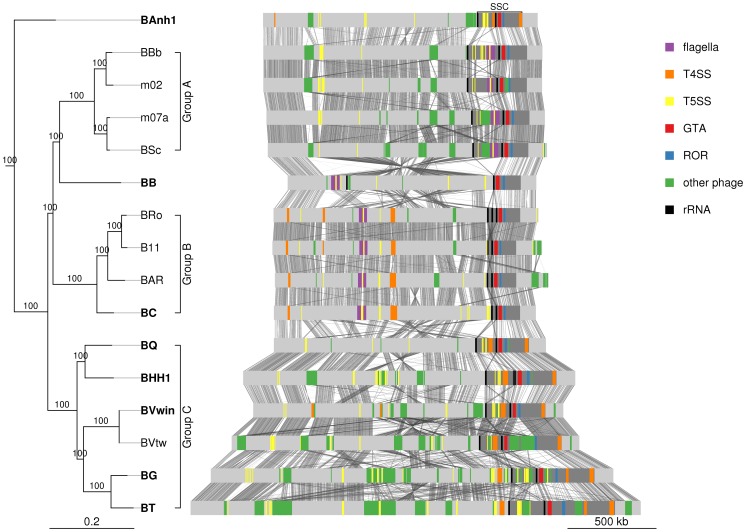
Comparative genomics of *Bartonella*. The *Bartonella* genomes are shown with grey bands and the location of secretion systems, rRNA genes and phage gene clusters are color-coded, as defined in the legend. Note that eight genomes have been closed (names in bold font: BAnh1, BB, BC, BQ, BHH1, BVwin, BG and BT) and that contigs of the other genomes have been mapped onto these (Figure S2). Tblastx hits between genomes are shown between the bands, with grey intensity reflecting the e-value of the hit. Abbreviations: SSC, secretion system cassette; T4SS, type IV secretion system; T5SS, type 5 secretion system; GTA, Gene transfer agent; ROR, run-off replication; rRNA, ribosomal RNA. The tree topology is as in Figure 1A and abbreviations of *Bartonella* species names as in Table 1. A similar figure with only T4SSs depicted is shown on Figure S9.

Previous studies of the *B. henselae* and *B. grahamii* genomes have shown that the gene cluster for the GTA is located in proximity to the peak of the amplification at the ROR region [9], [11]. Also located within the amplified segment are two rRNA operons (*rrn*) that flank a region containing type IV and type V secretion systems [9], [11]. These features are conserved in the *Bartonella* genomes examined here with the exception of *B. bacilliformis* where an inversion flanked by the 5′end of the second rRNA operon has translocated the entire SSC-segment to the symmetrical opposite side of the bacterial origin of replication. In all genomes including *B. bacilliformis* the gene cluster for BaGTA is located upstream of the ROR region (Figure 4), with a median distance of 32 kb and a maximum of 91 kb in *B. grahamii* (Table S6). The first rRNA operon is located further upstream of BaGTA, with a median distance of 197 kb from the ROR region (Table S6). Located at a median distance of 145 kb on the opposite side of the ROR region is the *trw* operon, coding for a type IV secretion system, in the group B strains and *B. australis*. We refer to this region, defined from the first rRNA operon to the *trw* operon and covering a median distance of 344 kb (Table S5), as the secretion system cassette (SSC) (Figure 4).

**Figure 5:**
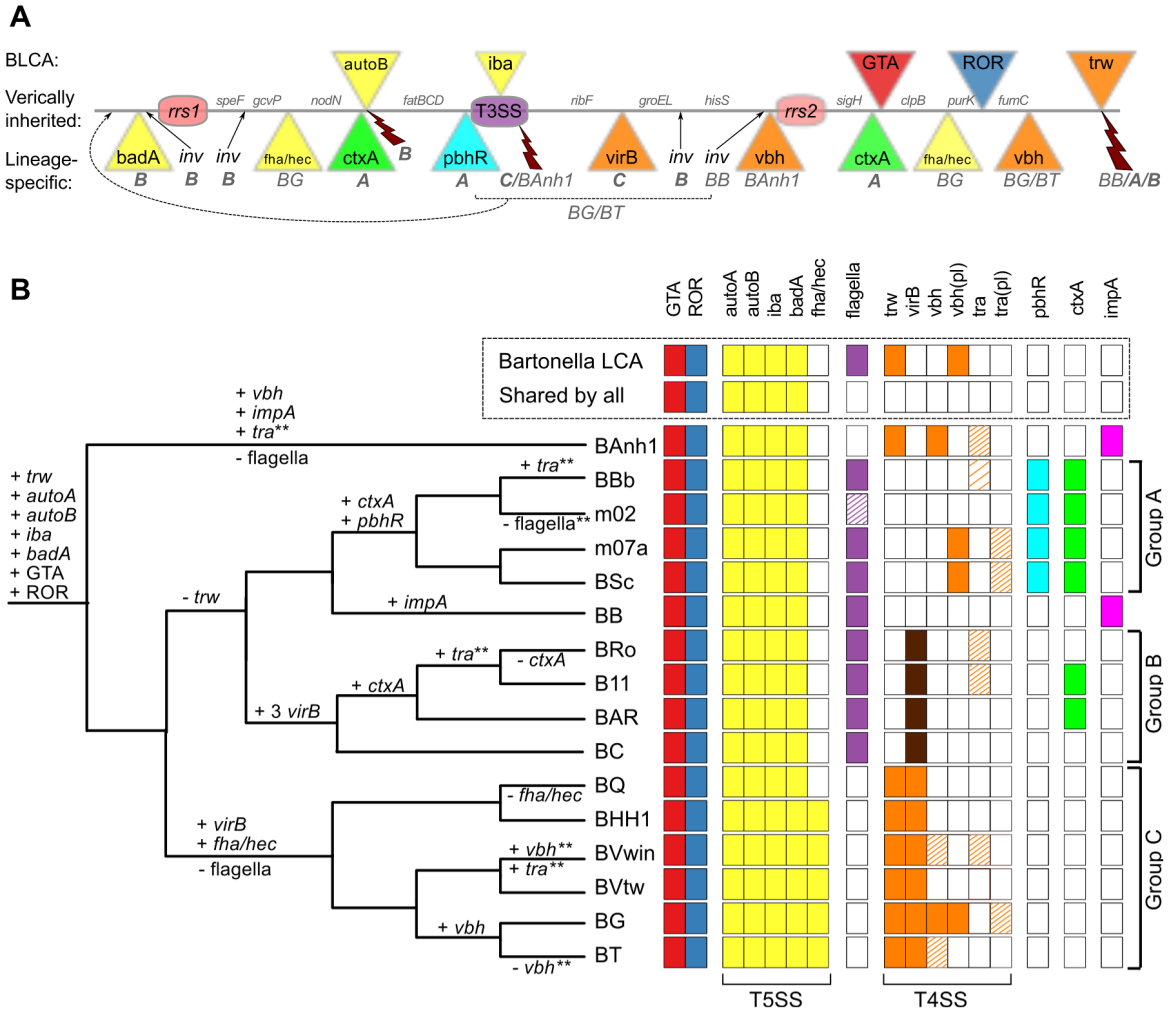
Genomic location and species distribution pattern of host-adaptation systems. The total set of identified gene clusters for secretion systems and toxins located in the vicinity of the gene transfer agent are shown in (A) and their presence/absence patterns among the *Bartonella* species analyzed here are shown in (B). The line in (A) represents the proposed ancestral chromosome. The genes inferred to have been acquired in the *Bartonella* common ancestor are shown above the line, and lineage-specific insertions below the line, with letters and species abbreviations referring to groups or species carrying the insertion. Broken arrows represent inferred deletions, with letters and species abbreviations referring to the groups or species carrying the deletion. (B) The presence/absence of each system in each genome is depicted as colored and white boxes, respectively and the inferred gains and losses are depicted above and below the nodes in the tree, respectively. Dashed boxes indicate that only presumably non-functional remnants are present. The density of the dashing reflects the amount of genes left intact in the system. Dark brown boxes in the *virB* column indicate multiple copies. * Partial loss. ** Partial gain, or gain followed by partial loss. The *vbh* plasmid [*vbh*(pl)] appears to be frequently gained and lost, and is omitted from the tree. Abbreviations: GTA, Gene Transfer Agent; ROR, Run-off Replication; LCA, Last Common Ancestor. The tree topology is as in Figure 1A and abbreviations of *Bartonella* species names as in Table 1.

We reasoned that ancestrally acquired genes that have been maintained in all genomes, like the BaGTA and the ROR described above, represent key innovative events that formed the basis for the explosive radiation of *Bartonella*. The identification of genes for secretion systems was particularly intriguing. In total, at least 8 gene clusters for secretion systems (*fha/hec*, *autoB*, *pbhR*, *fla*, *iba*, *virB*, *vbh*, *trw*) have been integrated into the SSC region (Figure 5A). We first set out to determine which of these gene clusters were acquired already in the common ancestor. Since several systems are encoded by duplicated genes and evolve very rapidly, they tend to be missed by automatic clustering of protein families. We therefore manually inspected all secretion systems and identified four type V secretion systems that are present in all *Bartonella* species (*autoA*, *autoB*, *iba*, *badA*), but not in the outgroup taxa (Figure 5B). Of these, the *iba* and *autoB* genes that code for autotransporters are located inside the SSC region.

Autotransporters represent a broad class of large proteins that contain a membrane-spanning autotransporter domain, which helps transporting the rest of the protein through the outer membrane [36]. The *iba* genes encode a protein called the inducible *Bartonella* autotransporter, which is represented by up to six tandemly duplicated gene copies. These genes have been shown to be upregulated during infection of endothelial cells *in vitro* [37], but it is not known whether they serve a role in cell adhesion. Interestingly, the *iba* genes have been inserted at the center of the flagellar gene cluster, consistent with ancestral presence of the flagellar gene cluster and a more recent acquisition of the *iba* genes. Despite being present in all genomes, the *iba* and *autoB* genes have undergone dramatic evolutionary changes, including both copy number variation and rapid sequence evolution, presumably driven by positive or diversifying selection to match a broad repertoire of host receptor molecules and/or to evade the host immune system. As such, they are likely to represent innovations that provided the foundation for the subsequent expansion into novel hosts. Based on the phylogenies and the conserved integration sites, we infer that the ancestral organization of the SSC region was: “-*rrn*1-core-*autoB*-core-*fla(iba)fla*-core-*rrn*2-core-BaGTA-core-ROR-core-” (Figure 5A).

### Expansion of host range and clade specific innovations

To establish a complete host-arthropod lifecycle, *Bartonella* must be able to attach to both nucleated host cells and erythrocytes [38]. Attachment to nucleated cells is mediated by autotransporter adhesins encoded by the *badA* genes [39], which are tandemly duplicated and located outside the SSC region, while the two gene clusters shown to be important for internalization into endothelial cells and for binding to erythrocytes, *virB* and *trw*, are located inside the SSC region in most genomes. Since host-specificity presumably resides in the sequences and structures of proteins that mediate these internalization processes, and since these are likely to have changed in response to an expansion or alteration of the host range, it is of interest to identify and characterize all clade- and species-specific gene acquisitions.

#### Acquisition of systems for infection of nucleated host cells

Studies of the internalization process have been done most rigorously in *B. henselae* [38]. These studies have shown that a few *Bartonella* cells first enter into vacuoles, where they start expressing *Bartonella* effector proteins (Beps) that are secreted through the VirB/VirD4 secretion system. The effector proteins blocks endocytosis, leading to the formation of bacterial aggregates on the host cell surface that is internalized *en masse* in the form of a so-called “invasome”. The *virB* gene clusters are present in both the A and the C-group *Bartonella* strains (Figure S9). V*irB* genes have been identified on plasmids in several other members of the Rhizobiales (Figure S10) and the phylogenies suggest that they have been horizontally transferred into *Bartonella*, followed by a functional switch. Moreover, it has been suggested that these systems have been acquired twice in the history of *Bartonella* and that their effector genes have been duplicated independently in groups B and C – which was surprising given the postulated sister relationship of these two groups [13]. However, our phylogenies suggest that groups B and C are not sister clades (Figure 1A), which supports the hypothesis that the *virB* systems were acquired independently in these two groups. This is also indirectly supported by the finding that the *virB* system is located inside the SSC region in all members of the C-group, but not in the B-group strain *B. clarridgeiae*, where 3 inversions have translocated the gene cluster coding for a flagellum and their flanking genes to the converse side of the chromosome [13].

The *vbh* gene cluster is a slightly divergent duplicate of the *virB* gene cluster, but only sporadically present in a few *Bartonella* species (Figure S10). Moreover, the *vbh* gene clusters are located on plasmids in *B. grahamii*, *B. schoenbuchensis* R1 and *B. schoenbuchensis* m07. Nearly identical copies of the *vbh* gene cluster on the *B. grahamii* plasmid were also identified in the *B. grahamii*, *B. tribocorum* and *B. vinsonii* chromosomes, although the clusters in the latter two species are in a process of deterioration. An independent chromosomal integration of the *vbh* gene cluster was observed in *B. australis*. The high variability among strains is consistent with transfers through plasmids, repeated chromosomal integrations at different sites and frequent losses.

#### Acquisition of systems for infection of erythrocytes

The *trw* gene cluster codes for the type IV secretion system Trw, which mediates host-specific attachment to erythrocytes [40]. Previous studies have shown that the *trw* gene cluster in the C-group strains has been horizontally acquired from a conjugation system similar to that on plasmid R388 in *E. coli* [41]. Here, we show that this system is also present in a single copy in *B. australis*. Unlike the *virB* system, which was acquired twice independently, we suggest that the *trw* system was acquired in a single horizontal gene transfer event. This is because it has integrated at the exact same site in all genomes, downstream of the BaGTA region in the SSC region. A shared common history is also confirmed by our phylogeny, which shows that the *trw* gene clusters of the C-group strains is a sister clade to that of *B. australis*, and that both are related to the *E. coli* plasmid R388 (Figure S10). By assuming ancestral presence, we have to further hypothesize that it was lost in the ancestor of the A and B-group strains (Figure 5B).

It has been suggested that the flagellar T3SS is involved in erythrocyte attachment in the A- and B-group strain that lack the *trw* system [38]. The hypothesis is that the gene cluster for the flagellum was ancestrally present and *trw* gene cluster was acquired to compensate for the loss of these systems in these two groups. Indeed, a phylogeny inferred from four proteins in the motor complex that drives the rotation of the flagellum and spans the cytoplasmic membrane, FlhA, FliI, FliP and FliQ, indicate presence by vertical descent (Figure S11). The presence of a gene cluster for the Trw system, but not for the flagellum in *B. australis* provides indirect support for the idea that these two systems are mutually exclusive. However, since *B. australis* represents the earliest diverging species in our set of genomes (or branches with the B-group strains that also do not have the *trw* genes), we have to infer a minimum of two independent deletion events to explain the observed presence/absence patterns. It is possible that both systems were ancestrally present and operated in parallel for some time before one of them took over the erythrocyte binding function. However, it should be emphasized that although the flagellum has been shown to be involved in host-interaction processes in many bacteria [42], [43], [44], [45], its role in *Bartonella* has as yet not been resolved [38].

#### Novel adhesin/secretion systems and cholera-toxin like genes in the A-group genomes

**Figure 6:**
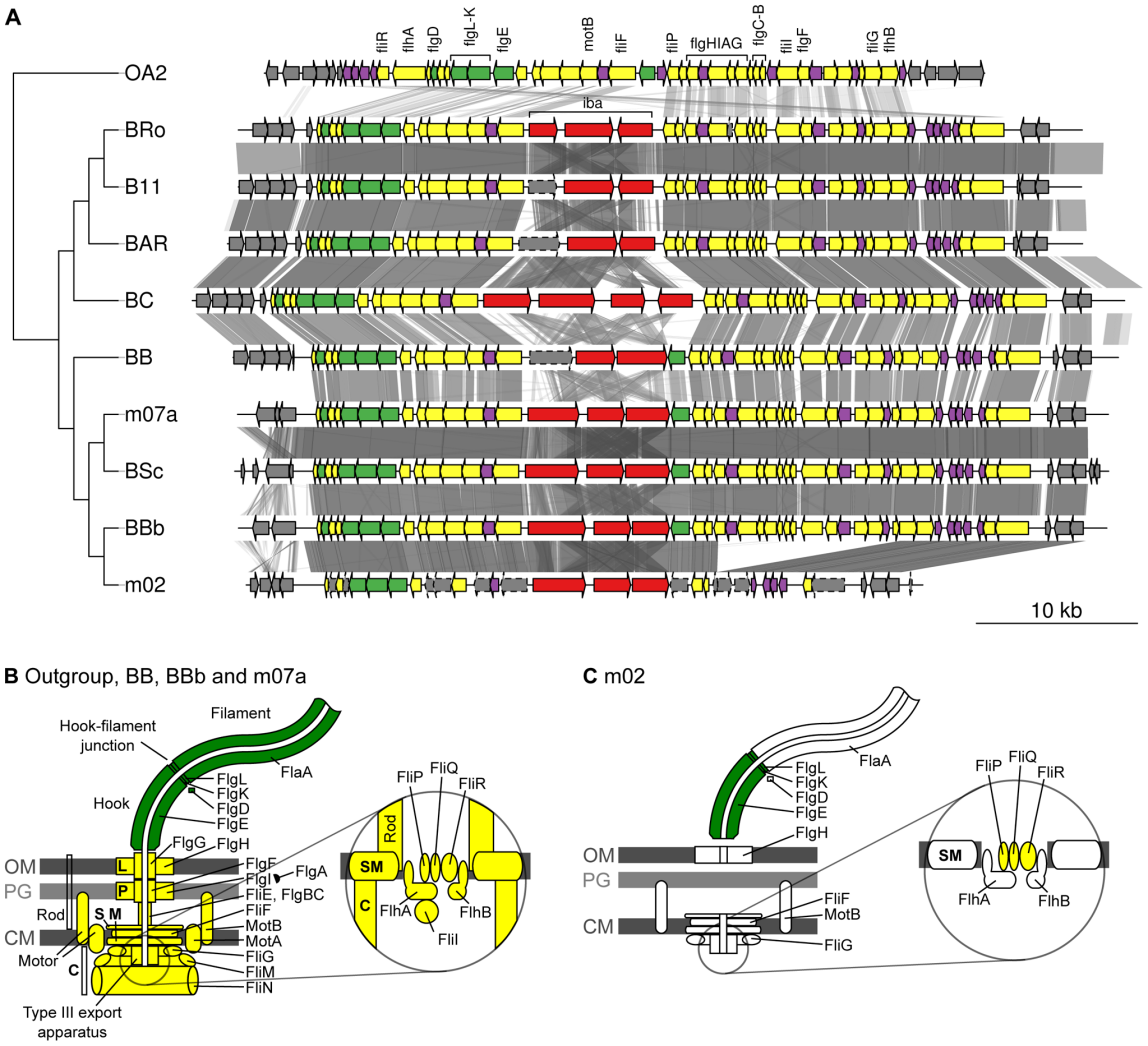
Gene order structures of the segment encoding the flagellum. Comparative analysis of the DNA segments covering the gene cluster for the flagellum (A). Tblastx hits between genomes are shown between the gene clusters, with grey intensity reflecting the e-value of the hit. The tree is a schematic representation of the phylogeny of the *Bartonella* species that contain a gene cluster for the flagellum, taken from Figure 1A. The outgroup OA2 is *Ochrobactrum anthropi* chromosome 2. Schematic representations of the flagellar protein complexes in species where the gene cluster is complete (B) and in *B. bovis* strain m02 where several genes are missing and other genes contain frameshift mutations (C). Genes and proteins are colored according to their presumed function and location: Extracellular proteins (green), membrane and intracellular proteins (yellow), hypothetical proteins (purple).

Given the demonstrated importance of the T4SSs for the infection process of *Bartonella* in mouse model systems [17], [19], it is remarkable that *B. bovis* strain m02 does not have a functional copy of either the *virB*, *vbh* or *trw* gene clusters, and only contains a very deteriorated flagellar gene cluster. Notably, a segment containing 15 flagellar genes has been deleted and another 10 genes have been pseudogenized in the *B. bovis* m02 genome, although all genes in the cluster are present in the closely related *B. bovis* 91-4 genome (Figure 6A, 6B). The deterioration process in *B. bovis* m02 has targeted 20 of the 27 genes for the core secretion system and the motility apparatus, whereas three of the five genes coding for the extracellular structures have been left intact (Figure 6C). Hence, other protein complexes than Trw or the flagellum have to mediate binding to erythrocytes in this strain.

Currently, the proteins mediating internalization into nucleated cells or binding to erythrocytes in the ruminant clade are unknown. But it is interesting to note that we identified a massively duplicated gene cluster in the A-group strains that cover an estimated 50–150 kb, precluding genome closure. This region is integrated at two major sites in each genome, one of which is located upstream of the *fla-iba-fla* gene cluster in the SSC region (Figure 5A). The segment contains a very complex repeat structure, with many tandem duplications and variably sized genes, indicating that it evolves under diversifying selection and may play a role in host-interaction processes. Provisional coding regions extracted from the draft assemblies revealed multiple copies of a parallel beta-helix repeat protein (PbH1; SMART accession number SM00710; [46]). For simplicity, we refer to the gene as *pbhR* for parallel beta-helix repeat domain. Intriguingly, this domain was identified in autotransporter proteins in several *Bartonella* species. The placement of one of the *pbhR* gene clusters in the SSC-region, the complex repeat-structures of the gene, the copy number variations and the apparent gene size heterogeneity resembles those of other T5SSs. We hypothesize that this novel gene family is a rapidly evolved variant of a T5SS, in which the autotransporter domain has been lost.

Equally interesting is the identification of a homolog to the *ctxA* gene for cholera toxin A in the A-group strains and in two of the B-group strains (E-value<10^−27^). The closest homolog to the *Bartonella ctxA* gene identified by BLAST searches was *Xanthomonas fuscans* subsp. *aurantifolii* ICPB10535 (acc. no. ACPY00000000) (E-value<10^−38^). In *Vibrio cholerae*, the toxin CtxA enters the host cell with the help of the smaller CtxB subunit (whose gene is located immediately downstream of *ctxA*) and then binds to an ADP-ribosylate adenylate cyclase, constitutively increasing the production of cyclic-AMP. In turn, this leads to the opening of ion channels and results in a massive efflux of ions and water [47]. This is interesting in the context of *Bartonella* since BepA, one of the VirB effector proteins, was recently shown to bind to the ADP-ribosylate adenylate cyclase, leading to an increased concentration of cAMP [48]. It was argued that the activation of the adenylate cyclase by BepA is subtle, consistent with the persistence of *Bartonella* in its natural host reservoir, in contrast to cholera toxin that cause acute and severe symptoms, and sometimes even death [47].

**Figure 7:**
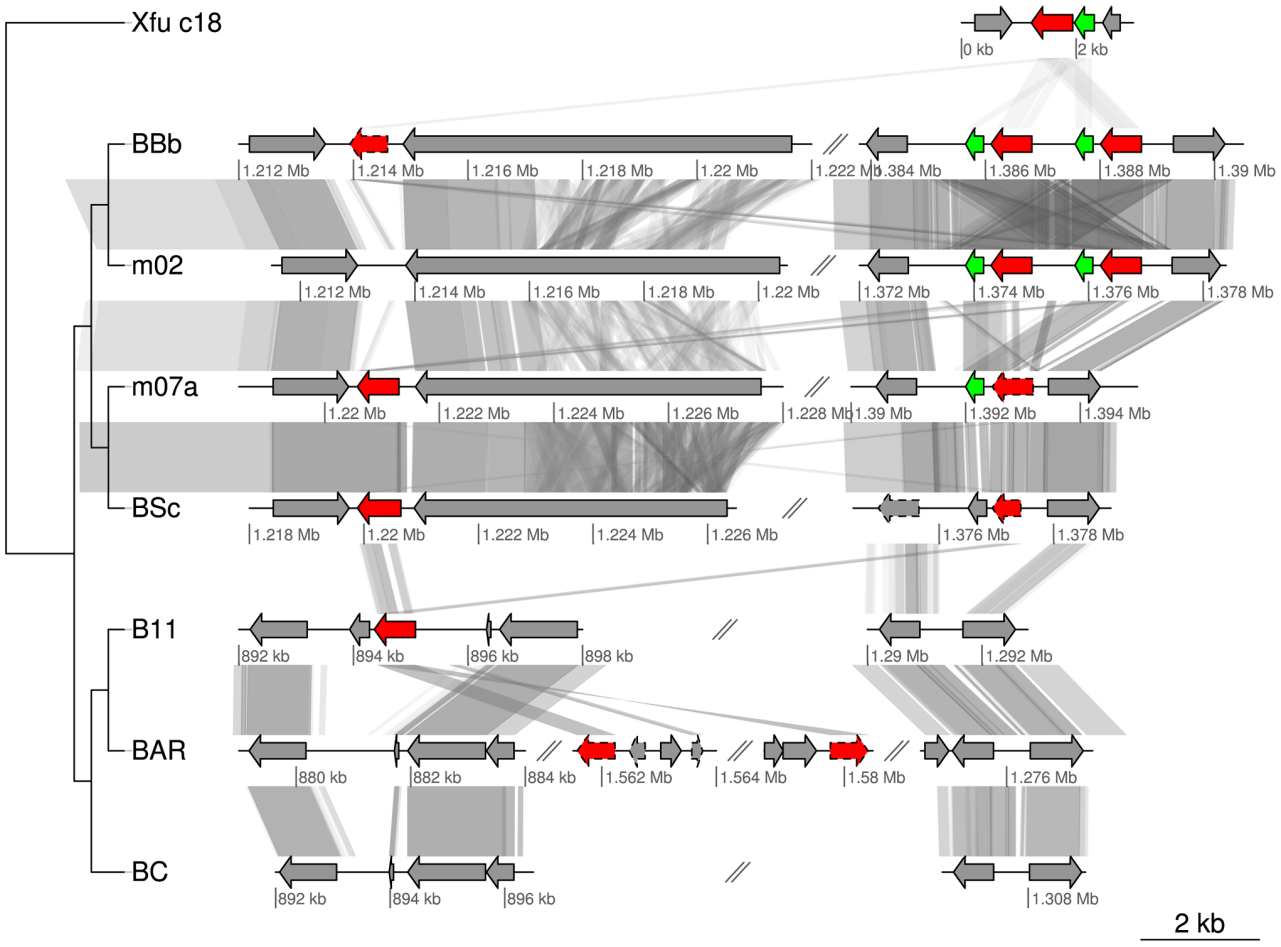
Gene order structures of the segment putatively coding for cholera toxin. Comparative analysis of the DNA segments covering the *ctxA* genes encoding cholera toxin. Homologs of the *ctxA* gene are marked in red and homologs to the gene upstream of *ctxA* in BBb are marked in green. Pseudogenes are marked with a dashed border. The first row is taken from contig 18 of *Xanthomonas fuscans* subsp. *aurantifolii* str. ICPB 10535 (NZ_ACPY01000017). Abbreviations of *Bartonella* species names as in Table 1.

Intriguingly, the *ctxA* gene homolog is present in the A-group strains that do not have the *virB* and the *bepA* genes, raising the possibility that they serve similar functions although they do not share any sequence similarity. It is also notable that while the *ctxA* gene is located in a prophage in *Vibrio cholerae*, it has integrated into the SSC region of the A-group genomes. There are one to two functional copies of the *ctxA* gene per genome in the A-group strains, and these are located either immediately upstream of the BaGTA gene cluster or immediately upstream of *autoB* (Figure 5, Figure 7). Moreover, there is a short gene downstream of the *ctxA* genes near to the BaGTA genes, which may correspond to *ctxB*, and these two genes are duplicated in tandem in *B. bovis* 91-4 and *B. bovis* m02. The *ctxA* gene was also identified in two of the B-group strains albeit at different genomic locations. Further experimental studies are needed to determine whether these proteins interact with adenylate cyclate and cause increased cAMP concentrations in the ruminant hosts.

#### A novel multi-copy gene family in *B. australis* and *B. bacilliformis*

In these comparisons, *B. australis* and *B. bacilliformis* are exceptional in that they have neither the *bep* nor the *ctxA* gene homologs. Although they are not sister species, both have a novel gene family in *Bartonella* that is present in about 15 copies in the *B. australis* and *B. bacilliformis* genomes, here referred to as the *impA* gene for “independently multiplied pathogen-associated gene”. The duplicated genes are organized into a major cluster of eight genes in *B. australis*, whereas the other 7 copies are located at multiple sites in the genomes. All copies within each genome cluster together in a phylogeny except for one of the *B. bacilliformis* genes that is associated with the *B. australis* clade, indicating independent multiplication in each genome (Figure S12). Homologs of these proteins have previously been identified in other human pathogens, and like in *Bartonella*, they are of unknown function and present in multiple copies in only a few species of the genus. For example, there are 13 copies in *Leptospira interrogans*, 3 copies in *Leptospira borgpetersenii* but none in their free-living relative *Leptospira biflexa*.

### Gene transfers

#### Horizontal gene transfer of secretion systems across the host species barrier

We argue that the advantage for a gene to be located within the amplified SSC region is that it increases the probability for recombination and spread of beneficial alleles within the population. This is indirectly supported by dramatically increased fixation rates for recombination events in the *virB* and *trw* gene clusters between strains in the *B. henselae* population [10], [34], but until now no direct gene transfers between strains naturally infecting different hosts have been identified. Interestingly, our study revealed such a case. The strain *B. vinsonii* Winnie was isolated from a dog that lived in the same household as eight cats, several of which were tested positive for *Bartonella* infections. Colony-forming units, identified as *B. henselae*, were obtained from two of the cats [22]. Although *B. henselae* could not be isolated from the blood of the dog, serological tests indicated that it had been infected with *B. henselae*.

**Figure 8:**
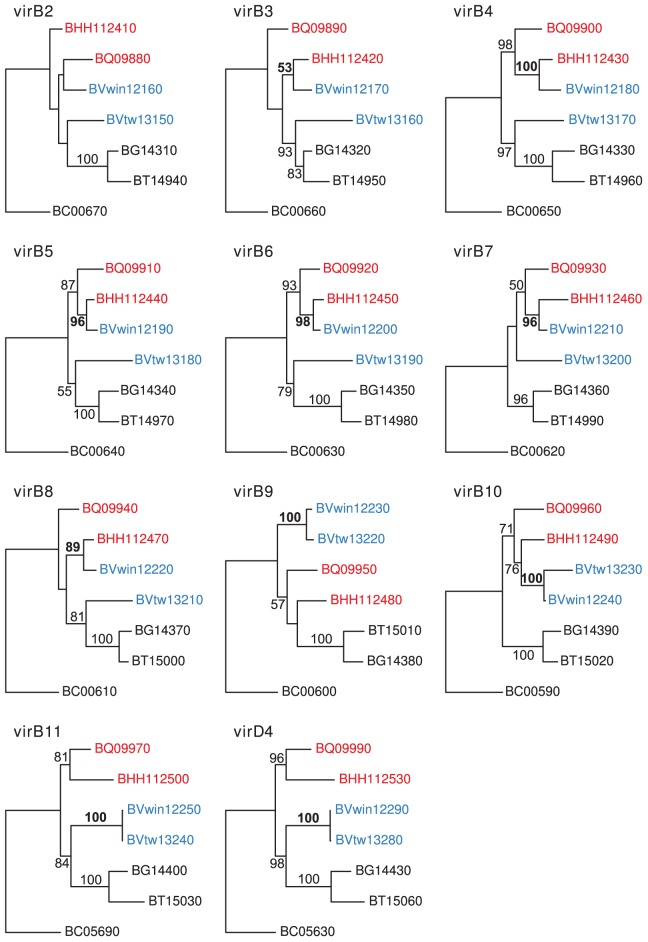
Horizontal gene transfer of the VirB genes across the host species barrier. Individual gene maximum-likelihood phylogenies for genes in the VirB cluster (*virB2*-*virB11* and *virD4*), based on DNA alignment of all species in the group C, using one of the homologs present in *B. clarridgeiae* as outgroup. Hundred bootstraps were calculated for each tree. The leaves are the locus ids of the genes in the cluster. Genes in *B. henselae* Houston-1 and *B. quintana* are shown in red, genes in *B. vinsonii berkhoffii* strains Winnie and Tweed are shown in blue, and genes in *B. grahamii*, *B. tribocorum* and *B. clarridgeiae* are shown in black. Bootstrap support higher than 50 is shown on the corresponding branches. The bootstrap support supporting the clustering of *B. vinsonii berkhoffii* Winnie with either *B. henselae* Houston-1 or *B. vinsonii berkhoffii* Tweed is shown in bold font.

Single gene trees based on seven genes in the *virB* gene cluster (*virB*2-*virB*8) showed that the dog-adapted strain *B. vinsonii* Winnie clustered with the cat-associated species *B. henselae*, rather than with *B. vinsonii* Tweed, the other isolate from a dog (Figure 8). The expected clustering of the two dog-adapted isolates was restored for genes located in the 3′-end of the clusters from *virB*9 to *virD*4. Thus, both the history of this strain and the phylogenies provide strong evidence to suggest that seven *virB* genes were transferred in a single event from a cat-adapted to a dog-adapted *Bartonella* strain. The transferred fragment was estimated to about 5 kb in size, small enough to have been transferred via the gene transfer agent.

#### Phage-mediated gene transfer of a kanamycin resistance gene

The comparative genomics study showed that a gene cluster for a gene transfer agent is present in the *Bartonella* genomes and also provided evidence that genes can be transferred among *Bartonella* strains under natural conditions. But is it the gene transfer agent that mediates these transfers? To test whether the GTA is functional, phage particles were isolated from the *B. henselae* strain Marseille31, which corresponds to mutant 31 in [49] and carries a transposon of 1221 bp carrying the Tn*903* kanamycin resistance gene inserted into a gene coding for a D-serine/D-alanine/glycin transporter. We isolated phage particles from the kanamycin-resistant Marseille31 donor strain and treated the phage particles with DNase prior to incubation with the kanamycin-sensitive *B. henselae* Houston-1 recipient strain. The resulting kanamycin-resistant Houston-1 cells were grown to visible single colonies after 13 days on plates containing kanamycin discs (Figure S13). The rate of efficiency of transfer was calculated from the number of resistant colonies divided by the number of recipient cells in the assay mixture. On average, the transfer rate was estimated to 2.5×10^−6^ transfers per recipient cell. Five kanamycin resistant colonies were shown to have the same MLST profile as the recipient Houston-1 cells and to contain a transposon insertion in their genomes. This shows that phage particles were able to transfer the transposon carrying the kanamycin resistance from one strain of *B. henselae* to the other.

### Selection hypotheses

We can think of two hypotheses to explain the linkage of gene clusters for secretion systems to the phage-derived origin of replication and the gene transfer agent: selection for regulation of gene expression or for enhanced recombination rate and enforced collaboration among cells. According to the regulation hypothesis, this may be a mechanism to regulate the level of transcription of the acquired secretion systems and surface proteins. Given the slow doubling time of *Bartonella*, the amplified genes could serve as templates for transcription before being degraded, packaged or recombined back into the genome. Such a process will result in an increase of the copy number that correlates inversely with the distance from the ROR region, and hence the mere placement of the gene could potentially influence its expression level. A rapid increase in gene copy numbers could be beneficial during invasion of host cells or at other critical life stages of *Bartonella*.

However, we consider such a regulatory scheme in its simplest form unlikely. First of all, a microarray study of expression changes in *B. grahamii* showed that although genes for secretion systems and phages located inside the amplified segment were upregulated, many core genes located in the same segment were not [9], arguing against a dosage effect. Moreover, copy number increases are expected to be similar for the *virB* and *trw* gene clusters for T4SSs, yet the *virB* gene cluster is expressed during invasion of endothelial cells [50], whereas the *trw* genes are expressed during invasion of erythrocytes [17], [40]. This does not preclude that increased copy numbers enhances the expression levels of some genes, but other regulatory signals must also be involved.

Alternatively, selection may act to increase levels of gene transfer and recombination of the secretion systems. Recombination can generate diversity that allows for rapid adaptation (to new hosts in the case of *Bartonella*), but is associated with the cost of disadvantageous alterations of core genes and disruptions of beneficial allele combinations [51]. With recombination targeting a specific chromosomal region, the positive aspects of recombination would be preserved, while the cost would largely be avoided.

**Figure 9:**
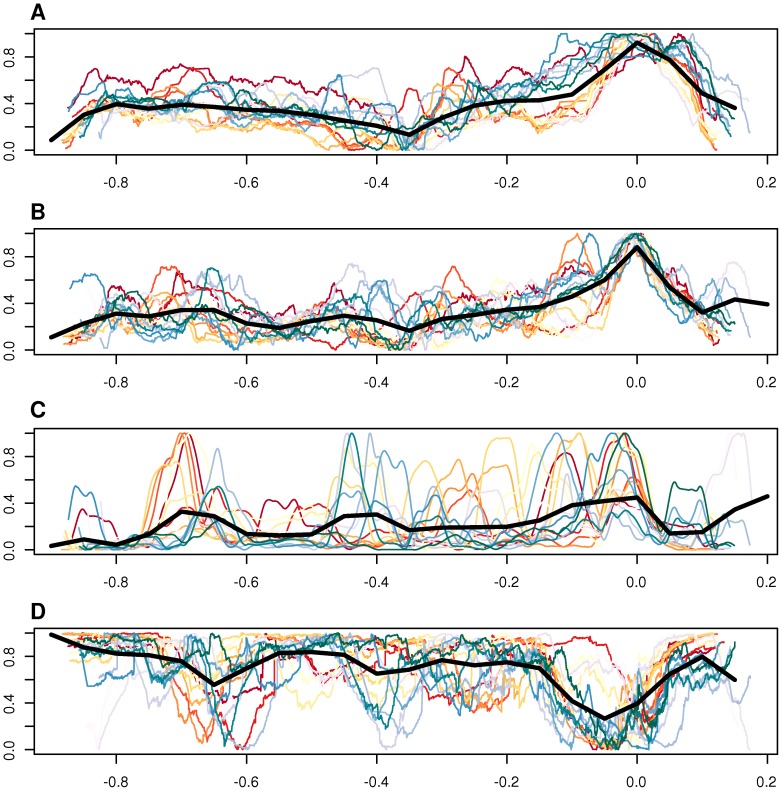
Comparative analysis of GC content, imported genes, and synteny blocks. Genomic features plotted against genome position, including GC content at the third codon position of all vertically inherited genes (A), GC content of intergenic spaces longer than 20 bp (B), frequency of imported genes in a 50-kb moving window (C), and synteny block conservation across species, weighted by the length of the block (D). Each colored line represent one of the 16 sequenced *Bartonella* genomes. The thick black line shows the median for all organisms, in a moving window. The size of the window is 1/20 of the total length of the x-axis. The x-axis shows the distance from the midpoint between BaGTA and ROR phages, normalized to the length of the genome. In the y-axes, the units in each panel have been normalized so that each line covers the whole interval [0,1], and the data has been smoothed with a third-order Savitzky-Golay filter, over 101 points (A–C) or with a weighted moving average over 501 points (D). On panel D, a value of 1 on the y-axis means that the segment is shared among all genomes.

There are several lines of evidence favoring this hypothesis. First, as shown by Touchon et al. [52], the amount of recombination correlates well with GC content at third codon position and we observed a gradual increase in GC content over the amplified region that follows the level of amplification with the peak located in the ROR region (Figure 9A, 9B). Second, it has been shown that DNA segments located in the amplified region are more frequently encapsidated into phage particles, increasing the likelihood for recombination with homologous DNA sequences in other bacterial cells [9]. Third, the higher GC content in the amplified region correlates with a higher abundance of imported genes (Figure 9C), although the increase in GC content is not restricted to the imported genes but also observed for core genes in the same region. Imported genes are here defined as genes present in any *Bartonella* strain, with homologs in distantly related bacteria but not in the outgroup species. Fourth, the fraction of synteny blocks shared among most or all *Bartonella* genomes is lowest in this region (Figure 9D). Finally, higher fixation rates for recombination events than in the genome overall has been demonstrated for several genes within the amplified region, including genes in the *virB* and *trw* clusters [10], [18]. Thus, the incorporation and maintenance of a gene transfer agent in the *Bartonella* genome may primarily be a mechanism to facilitate recombination and thereby speed up the evolution of adaptive traits.

Although the latter hypothesis is supported by the data and seductive at species level, it remains to be explained how it functions at the level of the individual bacterial cell since such a collaborative system is prone to cheaters in the population. This may not be a problem in the case of genes coding for surface components that are required for an individual bacterial cell to attach to a receptor on the host cell surface. In these cases, selection will act on the maintenance of these systems in every single bacterial cell rather than on the population as a whole. However, genes coding for proteins that are secreted into the host cytoplasm to modulate host cellular functions and pathways, such as the *bep* genes and possibly the *ctxA* gene homologs, should be prone to deletions in the individual cell. Secreted proteins are of importance for the population at large, but often costly to make for the individual cell. The population is therefore vulnerable to the emergence of cheaters who rip off the benefits without contributing to the production of the common good. This is particularly true for the *bep* genes, which are expressed by a few bacterial cells to block endocytosis, thereby making it possible for the large majority of cells, which may not necessarily need to have the *bep* genes, to enter the host cell through the formation of the invasome. Experimental studies have identified cheaters in bacterial populations that depend on the secretion of iron-scavenging molecules [53], digestive enzymes and quorum-sensing molecules [54] and antibiotic resistance factors [55], [56]. Despite the risk for collapse of the social trait, a dilemma referred to as “the tragedy of the commons” [57], [58] secretion is widespread in bacteria, not the least in host-adapted bacteria.

To solve this problem, mechanisms must exist that force selfish cells to cooperate. One strategy to enforce a cooperative behavior is to associate genes for secreted compounds with mobile elements, thereby ensuring a constant re-acquisition of the genes [59]. Indeed, genes for secreted proteins are overrepresented on mobile elements in 22 *Escherichia* and *Shigella* genomes [60]. Thus, the benefits of associating genes for secreted molecules with a GTA and a separate origin of replication could be several-fold: to generate variability and rapid spread of beneficial genes as well as to enforce collaboration among cells in the population. This could help explain why “social” genes coding for secreted effector proteins tend to be located inside the amplified SSC region, whereas genes for other surface molecules that mediate attachment and generate a benefit only to the bacterial cell in which the protein is produced may be located elsewhere.

### Concluding remarks

This study provides the broadest genomic study so far of vector-borne bacteria that infect mammals. Contributing 6 new *Bartonella* genome sequences to the previously sequenced 10 genomes, we identified a recently discovered gene transfer agent (BaGTA) and a phage-derived origin of replication as the major innovations in the last common ancestor of *Bartonella* that have since been maintained in all species. In contrast to a rapid turnover of other phage genes, the BaGTA and the phage-derived origin of replication are highly conserved in sequence and gene order and they are located at the same sites in all *Bartonella* genomes examined in this study, suggesting a single integration event. Previous studies of *B. grahamii* have shown that the phage-derived origin of replication drives the amplification of a surrounding chromosomal segment of several hundred kb, which is then digested into 14 kb DNA fragments and packaged into the phage particles [9]. In this study, we have extended these findings by demonstrating that the BaGTA can transfer genes between different strains of *B. henselae*. Because of the extreme conservation of the BaGTA and the associated origin of replication, it is reasonable to believe that the BaGTA serve as a gene transfer agent in all species examined here. Located within the amplified segment are many species- and clade-specific gene clusters for autotransporters and other secretion systems. In total, at least eight gene clusters for putative adhesins and secretion systems have been integrated into this region. Taken together, this suggests that the main role of the BaGTA is to shuffle genes for secretion systems located in this region among cells in the *Bartonella* populations.

We hypothesize that the plasticity given to this region by the BaGTA has rescued *Bartonella* from the reductive evolutionary processes that are operating on host-specialized bacteria adapted to otherwise sterile intracellular environments [61]. Previous studies have associated adaptive radiation in *Bartonella* with two independent acquisitions of the *virB* gene clusters for T4SSs along with genes for associated effector proteins [13]. Our tree topology suggests that the two clades containing these systems are not sister groups, which provides additional support to the hypothesis of two independent events. Experimental studies on the *virB* and *trw* gene clusters for type IV secretion systems have shown that they are essential for the infection of endothelial cells and erythrocytes, respectively. Amplification and recombination of this region facilitates duplication and functional divergence, contributing to the generation of a diverse set of proteins that can bind to a variety of host cell molecules in a broad range of hosts.

This organization may also facilitate reintroduction of the secretion systems into cells and populations that have lost them. Since secretion systems and adhesins are outer surface proteins that may be recognized by the host immune system, selection may favor downregulation or loss at some stages during the infection. For example, in regions where hosts and vectors are abundant and the propensity for super-infections is high it may be beneficial to shed or modulate some of these genes. We observed here that one of two closely related moose isolates have lost most of the genes for a flagellar type III secretion system. Likewise, we have shown previously the presence/absence patterns of genes for filamentous hemagglutinin (a type V secretion system), differs in mouse-infecting *B. grahamii* populations sampled from geographically close locations [62]. An additional benefit is that repeated reintroduction of these genes provides a mechanism that forces all cells in the population to contribute to the production of the secreted molecules. More detailed studies of the evolution of these systems during different stages of the infection in individual hosts could help clarify the interplay between secretion systems, infection and immunity.

The results presented in this paper contribute to a greater general understanding of the evolutionary significance of gene transfer agents and their role in driving adaptive radiation processes. The identification of BaGTA is remarkable given the prevalence of RcGTA-like gene clusters in most other Alphaproteobacteria, including genera such as *Brucella* and *Agrobacterium* that are closely related to *Bartonella*. The two GTAs are not related, suggesting that BaGTA has replaced RcGTA. Features associated with BaGTA, such as the phage-derived origin of replication and the amplification has not been identified in genomes that host the RcGTA. Future analyses of conserved phage elements will be needed to determine whether the association of gene transfer agents with adaptive traits is a common theme in bacterial genomes.

## Materials and Methods

### Bacterial strains

The type strains *Bartonella australis* NH1, isolated from a grey kangaroo [20], and *B. bovis* (Bermond) 91-4, isolated from a cow [21] were obtained from the Culture Collection at the University of Göteborg, Sweden (CCUG numbers 51999 and 43828, respectively). The strains *Bartonella vinsonii berkhoffii* Winnie [22] and Tweed [23], isolated from dogs, were obtained from Ricardo Maggi and Edward Breitschwerdt (Department of Clinical Sciences, College of Veterinary Medicine, North Carolina State University, Raleigh, USA). The strain *Bartonella bovis* m02, isolated from a moose in Sweden, was obtained from Martin Holmberg (Department of Medical Sciences, Uppsala University, Uppsala, Sweden). The strain *Bartonella schoenbuchensis* m07a was isolated from a blood sample collected from a moose close to Dyltabruk, Örebro county, Sweden, on October 28, 2007. The sample was cooled after collection, and stored at −80°C for 30 days before cultivation on hematin agar plates in a humidified 5% CO_2_ incubator at 35°C for 5–7 days.

### Genome sequencing and analysis

#### Genome sequencing

Starting from single colonies, all strains were grown on hematin agar plates for five days. Bacteria from each plate were collected with the help of sterile cotton applicators and suspended in 500 µl of phosphate-buffered saline solution. After centrifugation, DNA was isolated with the AquaPure DNA Isolation Kit (Bio-Rad, Hercules, CA) according to the manufacturer's instructions. The precipitated DNA was resuspended in 100 µl of DNA hydration solution (Bio-Rad) to a final concentration of 300 to 1,000 µg/ml per plate. Sanger sequencing was performed on an ABI 3730XL (Applied Biosystems, Foster City, CA, USA). Pyrosequencing was performed with 454 GS20, FLX or Titanium chemistries (Roche/454 Life Sciences, Branford, CT, USA) at the KTH Sequencing Facility, Royal Institute of Technology, Stockholm Sweden. Illumina was done on a Genome Analyzer II (Illumina, San Diego, CA, USA) at Fasteris SA in Geneva, Switzerland. The GS FLX Titanium 20-kb paired-end library (Roche/454 Life Sciences, Branford, CT, USA) was prepared according to the manufacturer's protocol. See Table S1 for a detailed summary of the used sequencing technologies.

#### Assembly and gap-closure

Assembly and gap-closure was performed using a combination of shotgun sequencing (Sanger and pyrosequencing), 20 kb insert library end-sequencing (pyrosequencing), fosmid library end-sequencing, long-range PCR, and Pulsed Field Gel Electrophoresis (Table S1). The assembly programs GS De Novo Assembler (Roche/454 Life Sciences, Branford, CT, USA), MIRA3 [63] and phrap (Phil Green, Genome Sciences Department, University of Washington, distributed by the author) were used, and manual editing was performed with Consed [64]. PCR products and fosmids were shotgun sequenced (Sanger) and assembled separately with phrap before being added to the main assembly. Contigs were concatenated based on paired-end and PCR information. Illumina reads were mapped to the genome assemblies using MIRA [63] to correct homopolymer errors. Dubious sites identified by MIRA were manually screened.

#### Alignment of draft genomes

For the purpose of whole-genome analyses, contigs in draft genomes were aligned to a reference genome with the MUMmer package [65], using nucmer with default settings and the show-tiling utility to find the best mapped location of each contig. For the latter, the genome was assumed to be circular, no maximum gap size or minimum contig size was set, the minimum identity was set at 40% and the minimum coverage at 30%. Contigs of *Bartonella rochalimae*, *Bartonella* sp. 1-1C and *Bartonella* sp. AR 15-3 were aligned to the chromosome of *B. clarridgeiae* and contigs of *B. bovis* m02, *B. schoenbuchensis* m07a and *B. schoenbuchensis* R1 were aligned to the scaffold of *B. bovis* 91-4.

#### Annotation

All 16 genomes, including the previously published genomes, were annotated using the same set of programs, to obtain a uniform annotation. DIYA pipelines [66] were created to automate as many steps of the process as possible. *De novo* gene predictions were performed for CDSs with prodigal 2.0 [67], rRNAs with RNAmmer 1.2 [68], and tRNAs with tRNAscan-SE 1.23 [69]. GenePRIMP [70] was then used to identify frameshifted genes: tandem CDSs with hits to the same gene were flagged as potential artificial frameshifts due to homopolymer errors, inspected manually and corrected whenever applicable. GenePRIMP annotations were also used to correct the position of start codons, wherever a majority of blast hits pointed to genes with different start sites.

CDS annotations were based on blastp 2.2.24 [71] searches to the published *Bartonella* genomes and to the nr database. The annotation of the *B. grahamii* genome was manually checked and corrected, such that it could be used as a reference annotation for the other genomes. The annotations of the CDSs, which belong to single gene family that includes all *Bartonella* species, were transferred from the *B. grahamii* annotation to the other genomes. All other CDSs were manually and individually inspected using Artemis [72] and an ad-hoc Perl interface. Annotation of CDSs that are part of host-interaction systems and phages were checked manually for consistency using ACT [72]. An overview of the annotation process is depicted in Figure S3.

#### Protein family clustering

All-against-all blastp [71] searches were performed with all CDSs from the 16 *Bartonella* genomes and the six outgroup species, with an E-value cutoff of 10^−3^. Blast hits covering less than 80% of the shortest CDS involved were discarded, as well as hits where one CDS was shorter than 60% of the other. To attenuate the effect of recent duplication events, intra-genomic blast hits were discarded if their bit score was smaller than any of their between-genome hits, as described in [73]. The CDS were clustered into families of protein homologs, using the MCL algorithm [26], which uses a hidden Markov model where the nodes are the genes and the edges the bit score values of the blast results. The parameters were set so that both orthologs and paralogs were generally included in the same protein family.

#### Data deposition

All reads involved in this study have been deposited to NCBI's Sequence Read Archive under study number SRP008782. Complete genome sequences of *B. australis* NH1 and *B. vinsonii berkhoffii* Winnie are deposited in Genbank under accession numbers CP003123 and CP003124, respectively. Whole-genome shotgun assemblies of *B. bovis* 91-4, *B. bovis* m02, *B. schoenbuchensis* m07a and *B. vinsonii berkhoffii* Tweed are deposited in Genbank with accession numbers AGWA00000000, AGWB00000000, AGWC00000000 and AGWD00000000, respectively.

### Molecular evolutionary analyses

#### The species tree

Two different dataset were used: the ingroup dataset consisting only of the 16 *Bartonella* genomes and the full dataset, which contains the 16 *Bartonella* genomes and the 6 outgroup species: *Brucella melitensis* (NC_003317-18), *Ochrobactrum anthropi* (NC_009667-72), *Mesorhizobium loti* (NC_002678-79, NC_002682), *Sinorhizobium meliloti* (NC_003037, NC_003047, NC_003078), *Agrobacterium tumefaciens* (NC_003062-65), and *Bradyrhizobium japonicum* (NC_004463).

Genes were defined as core and included in the analysis if they were present as single copy genes in all 22 genomes. For the phylogenetic analyses, this set was supplemented with the ribosomal protein genes, which are duplicated in *B. bacilliformis*, as well as the house keeping genes *groEL*, *groES* and *gltA*, which are duplicated in the outgroup species. The alignments were trimmed down to contain only one gene per taxon, keeping the copy with the least divergence from the sequences of the other taxa. This resulted in a dataset of 428 core genes. All genes were aligned using MAFFT 6.710b (L-INS-I) [74], and poorly aligned regions were filtered away with Gblocks [75]. For the species tree, the alignments of the 428 core genes were concatenated into a single alignment using in-house Perl scripts. Subsets were prepared in which the alignments of genes with similar divergence levels were concatenated (average pairwise divergence levels between all sequences within the alignment).

Maximum likelihood phylogenies were inferred using RAxML-VI-HPC 7.0.4 [76], with the GTRGAMMA model for nucleotide and the predicted model of ProtTest2.4 [77] for the amino acid data and 100 bootstraps. Bayesian phylogenies were inferred using Phylobayes 3.2f [78], using a GTRCAT model, with a continuous gamma distribution of site variation. Five chains were run and checked for convergence before stopping them. Tree topology tests were performed using Shimodaira-Hasegawa (SH) likelihood comparison tests [79] as implemented in *Baseml* and *Codeml* in PAML 4.0 [80]. These tests allow comparing different tree alternatives using their log-likelihood values computed with different models, given an alignment. This method was used for testing the placement of the position of the root of the *Bartonella* clade, as well as the position of *B. australis* within the *Bartonella*. The seven tested root position are shown in Figure S6 and the five tested placements of *B. australis* are shown in Figure S4. The previously published *B. australis* sequences (DQ538394-9) were aligned to their respective *Bartonella* homologs in the sequenced genomes, and maximum likelihood phylogenies were inferred as above.

#### Discordance filter

A core gene dataset was obtained as explained above, including the genes *Bartonella tamiae* Th239, for which a whole-genome shotgun experiment was recently deposited in Genbank (accession number AIMB00000000). The dataset consists of 423 protein families. The sequences were aligned and the alignments filtered as above. For each alignment, 100 bootstraps were calculated using RAxML-VI-HPC 7.0.4 [76], using the PROTCATWAG model. A discordance filter was then applied, as in [28]. Shortly, among the bootstraps, splits or bipartitions supported by less than 75 bootstraps were discarded. A pairwise conflict score for each pair of family was calculated as the number of remaining incompatible splits in both split sets divided by the product of the number of splits in both sets. The discordance score of each family was defined as the sum of the family's conflict scores with each other family. The families were then ranked by their discordance score. Concatenated alignments of gene families were built, removing an increasing percentage of the most discordant families. For each of these concatenated alignments, a maximum likelihood phylogeny was inferred with RAxML-VI-HPC 7.0.4 [76], using the PROTCATWAG model, and 100 bootstraps were calculated. From each bootstrap set, the bootstrap support for four critical splits was extracted. The critical splits considered were: *B. australis* NH1 clustering with the outgroup including *B. tamiae* (“BAnh1 first”), or with group B species (“BAnh1 with B”); *B. bacilliformis* clustering with the outgroup including *B. tamiae* (“BB first”), or with group A species (“BB with A”).

#### Single gene trees

The flagellar gene phylogenies were inferred based on four flagellar genes (FliI/SctN, FlhA/SctV, FliP/SctR, FliQ/SctS), including their T3SS homologs. We used the same protein sequences as in [81], which were selected to represent the diversity of the flagellum and the T3SS, and added homologs in the *Bartonella* genomes and the six outgroup species. Homologs were detected by PSI-BLAST, using the flagellar genes from *B. bacilliformis* and the T3SS genes from *Mesorhizobium loti* as queries, with an E-value cut-off of 10^−4^. The datasets were manually inspected, resulting in a final dataset of 61–65 genes. The *virB4* phylogeny was inferred using the same protein sequences as in [41], to which we added homologs from *Bartonella* genomes and the six outgroup species. The protein sequences were aligned by MAFFT 6.710b (L-INS-I) [74] and phylogenies were inferred using maximum likelihood with RAxML-VI-HPC 7.0.4 [76], substitution model PROTGAMMAIWAG (predicted by ProtTest2.4) [77] and 100 bootstrap replicates.

Sequences for genes in the VirB cluster (*virB2-virB11* and *virD4*) were retrieved for all species in group C and for their first homolog in *B. clarridgeiae*, as an outgroup. DNA sequences were aligned with MAFFT 6.710b (L-INS-I) [74], and maximum-likelihood phylogeny was inferred using RAxML-VI-HPC 7.0.4 [76], with substitution model GTRGAMMA and calculating 100 bootstrap replicates.

The *impA* phylogeny was inferred using all identified homologs by iterative PSI-BLAST [71] searches in the nr database and the draft *Bartonella* genomes, with an E-value cut-off of 10^−4^. In total, 65 genes were retrieved, 15 in *B. bacilliformis*, 17 in *B. australis* NH1, one in *Helicobacter hepaticus* ATCC 51449, one in *H. mustelae* 12198, three in each of the sequenced *Leptospira borgpetersenii* serovar Hardjo-bovis (strains L550 and JB197), and 13 in each of the sequenced *Leptospira interrogans* (serovar Lai str. 56601 and serovar Copenhageni str. Fiorcruz L1-130). The resulting protein sequences were aligned with MAFFT (L-INS-I) [74]. A phylogenetic tree with posterior probability estimates was inferred with MrBayes 3.2 [82], using the WAG+I model [83], 10 million generations and a burn-in of 1 million generations. In addition, 100 bootstrap replicates were run with RAxML [76], using the WAG model.

#### Substitution frequencies

To test for the uniformity of substitution frequencies in the BaGTA and ROR and around, the BaGTA and ROR region in the genomes belonging to groups A, B and C were visually inspected in ACT [72], and intra-lineage positional orthologs were identified. Five regions were determined: the BaGTA (11–13 genes), the ROR (6–7 genes), and three region encompassing core genes, the spacer between them (9–12 genes), the region between the ribosomal RNA operon and BaGTA (9–12 genes), and a region located downstream of the ROR (10 genes). For each of these regions and lineages, pairwise substitution rates were calculated with the yn00 utility implemented in PAML 4.0 [80]. The intra-lineage substitution frequencies were averaged for each region. A Welch two-sample t-test was performed to compare both the BaGTA and ROR genes to the pooled core regions.

#### Identification of repeats

To identify repeated structure similar to the one found in the O-protein gene of the lambda phage, genes of the ROR phage were screened with REPuter [84]. The settings were tested on lambda sequence, so as to detect the structures described in [35]. The sequences were searched for exact forward repeats of at least 8 nt, and for palindromic repeats of at least 16 nt, with at most 4 edits.

### Gene flux analysis

#### Ancestral reconstruction of protein families

All changes in protein families defined by the MCL algorithm (see above) were mapped on the two most probable species tree, without branch lengths, including the 16 *Bartonella* genomes and the six outgroup species, with the tree rooted based on previous results [33], [34]. Gain and loss of gene copies in each protein family were reconstructed over the tree using generalized parsimony with ACCTRAN in PAUP* 4.0b10 [85], using a cost matrix with the following weights; gene genesis = 10, gene loss = 5, gene duplication = 1, and all other copy-number variations = 0.2 per copy.

Using the ancestral reconstruction, CDSs were attributed to four phylogenetic categories (vertically inherited, imported, *Bartonella*-specific and ORFans). Gene families present in the *Bartonella* last common ancestor (BLCA, node 39 in Figure 2A) and its parent node in the tree (node 41 in Figure 2A) were designated as vertically inherited. To classify the rest of the families, a PSI-Blast search of the genes to the nr database was performed. If it resulted in one or more hits with an e-value<10^−10^, or three or more hits with an e-value<10^−6^, the family was classified as imported. For the remaining protein families, those present in BLCA were categorized as *Bartonella*-specific, the rest as ORFans.

#### Gain and loss of host adaptation systems

All potential host adaptation systems were manually inspected in each genome, and their chromosomal locations were manually evaluated by visual genome comparisons using ACT release 9 [72]. The inference was guided by the idea that losses occur more easily than gains. For the T4SSs, we generally assumed independent gains to be more likely than a single gain followed by specific relocation of a complete system to a new chromosomal neighborhood.

### Experimental analysis

#### Infection assays

The isolate *B. bovis* m02 was grown on 5% bovine blood agar plates (SVA, Uppsala, Sweden) at 35°C in a humidified atmosphere of 5% CO_2_. For infection assays, bacteria harvested from 4 days grown plates were suspended in cell culture medium (see below) and adjusted to the appropriate concentration.

The bovine aortic endothelial cell line GM07373 (BAEC), obtained from the Coriell Cell Repository (Camden, NJ, USA), was cultured in Minimum Essential Medium (MEM) with Earle's salts supplemented with 10% fetal bovine serum (FBS), 1 mM non-essential amino acids, 100 U/ml penicillin and 100 µg/ml streptomycin at 37°C in a humidified atmosphere of 5% CO_2_. Subconfluent cells grown in 12-well culture plates (Nunc), and on coverslips placed inside individual wells, were infected with a multiplicity of 100 CFU per cell in MEM containing 10% heat-inactivated FBS and 1 mM non-essential amino acids. Bacterial adherence to the cells was stimulated by a brief centrifugation (100 g, 5 min). Infected cell cultures were maintained at 37°C in a humidified atmosphere of 5% CO_2_ with daily replacement of the medium.

Infected cells were harvest by trypsin-EDTA treatment, centrifuged, washed in PBS and fixed in 2.5% glutaraldehyde buffer for several days at 4°C. Post fixation was for 45 min in phosphate-buffered 1% osmium tetroxide, followed by washing with PBS under low speed centrifugation. The resulting pellets were placed in agar, cut into small blocks and dehydrated in sequentially graded ethanol. After acetone dehydration, blocks were embedded in TAAB 812 epoxy resin and sections for TEM were cut at approximately 50 nm using a LKB Ultramicrotome. The Ultrathin sections were stained with uranyl acetate for 30 min followed by lead citrate for 10 min and examined with a Zeiss Supra 35-VP (Carl Zeiss, Germany) field emission SEM equipped with a STEM detector for transmission microscopy.

*In vitro* invasiveness was examined at different time points post infection (p.i.) by AO-CV staining as described by Miliotis [86]. In brief, BAECs cultured on coverslips were washed two times with PBS and, without fixation, stained with 0.01% acridine orange in Gey's solution for 45s, followed by counterstaining with 0.05% crystal violet in PBS for 45s. The coverslips were then rinsed with Hanks balanced salt solution, mounted on slides and sealed with colorless nail polish. Slides were examined by fluorescence microscopy at 100× magnification using a Leica DMRXE microscope (Leica Microsystems).

#### Transduction of a kanamycin-resistance gene

In brief, phages were purified from cultures of kanamycin-resistant donor cells and incubated with kanamycin-sensitive recipient cells of a different sequence type. Transduced cells were grown on kanamycin plates. The genotypes of resistant cells growing on the plates were determined by sequencing PCR products obtained with MLST primers to differentiate between recipient and any potentially remaining donor cells.

*B. henselae* Marseille31 [49], which contains a transposon of 1221 bp carrying the Tn*903* kanamycin resistance gene (EZ::TN <KAN-2> Tnp Transposome Kit, Epicentre Technologies, Madison, WI, US) inserted in a gene coding for a D-serine/D-alanine/D-glycine transporter (kindly provided by Volkhard Kempf, Germany) was used as the donor cells. Kanamycin-sensitive *B. henselae* Houston-1 (ATCC 49882) was used as recipient cells. Donor and recipient cells were separately grown for 5 days at 35°C in a humidified atmosphere of 5% CO_2_ on 5% bovine blood agar plates (SVA, Uppsala, Sweden). Cells were harvested with a sterile cotton swab and resuspended in a phosphate-buffered saline (PBS) solution, and then centrifuged at 3000 rpm for 3 minutes. Liquid cultures were performed under agitation (∼80 rpm), at 35°C in a humidified atmosphere of 5% CO_2_, in pre-warmed pH-balanced liquid medium (PBLM): 24.5 g/l Schneider's insect medium with glutamine (Sigma-Aldrich, St. Louis, MO, USA), 5% (wt/vol) sucrose, 0.025 M HEPES, 5% heat-inactivated fetal bovine serum (Gibco, Carlsbad, CA, USA), equilibrated at pH 7.2 [87]. Recipient cells were grown in liquid culture as above, starting with 10 ml PBLM Cells were harvested at OD_600_ = 1.0 and resuspended in PBS at OD_600 = _1.5.

To isolate phages, a liquid culture was started by inoculating donor cells at OD_600_ = 0.01 in 25 ml PBLM, adding 30 µg/ml of kanamycin. Cells were harvested at OD_600_ = 0.6 by centrifugation (3200 rpm, 20 minutes at 4°C). The supernatant was filtered on 0.2 µm sterile filters and precipitated on ice at 4°C by adding a volume corresponding to one third of the filtrate of a solution consisting of 20% polyethylene glycol 8000 and 2.5 M NaCl. Phages were centrifuged (12'000 rpm, 45 min at 4°C) and resuspended in 60 µl PBS, of which 9 µl was mixed with 1 µl of 1 unit/µl DNase (Sigma) and incubated for 30 minutes at room temperature.

To perform transduction, 100 µl of the recipient cells resuspension was mixed with 2 µl of the phage solution, and incubated for 2 hours at 35°C under humidified atmosphere of 5% CO_2_. After incubation, the mix was plated on bovine blood agar plates, with kanamycin discs (30 µg, Difco, Franklin Lakes, NJ, USA). Plates were incubated for 13 days in the same conditions as above. To test for the absence of donor bacteria in the phage solution, the same procedure was repeated, omitting the recipient cells. No colonies were observed on the plates where only the phage solution was plated. Sequence data was collected from five kanamycin-resistant colonies, following a MLST scheme [11]. Only loci that were different in the two strains (Houston-1 and Marseille were tested, namely *rrs*, *batR*, *ftsZ*, *gltA*, *groEL*, and *rpoD*. Resulting sequences were compared to the reference strains published in [11].

### Figures

Figure 3, Figure 4, Figure 6A, Figure 8, Figure S2, and Figure S10 were drawn with genoPlotR 0.7 [88]. Figure 9 was obtained with R 2.12.1 [89]. All trees were drawn with FigTree (Andrew Rambaut, available on the author's website: http://tree.bio.ed.ac.uk/software/figtree/).

## Supporting Information

Figure S1 Infection of endothelial cells by *Bartonella bovis* m02. Acridine orange staining (upper panels) and transmission electron microscopy (lower panels) of bovine aortic endothelial cells both during uninfected conditions (left panels) and at 72 hours post infection (right panels) with *B. bovis* strain m02. The intracellular bacterial aggregates are visible as green dots around the nucleus (upper right panel) and as dark spots inside light grey vacuoles (lower right panel). The scale in each electron micrograph is indicated by a black bar (1 µm).
(TIFF)

Figure S2 Map of contigs and scaffolds in the 16 *Bartonella* genomes. Complete and closed genomes are represented as blue lines, draft genomes as red arrows. Each arrow represents a contig. Scaffolds are separated by “//”. In *B. bovis* 91-4 and m02, *B. schoenbuchensis* m07a and *B. vinsonii berkhoffii* Tweed, the contig that spans the origin of replication has been broken up, resulting in a linear representation of the rightmost contig in the genome. Contigs that have not been placed within the scaffolds and that do not map to the genome of comparison are added at the right of the linear representation of the genome. The tree topology and the genome scale bar are as in Figure 4. The pairwise comparisons are as in Figure 4, except that direct hits are represented in red and complementary hits in blue.
(PDF)

Figure S3 Overview of the annotation process. Blue background in boxes represent input data and green color a step in the pipeline. Boxes surrounded by larger orange boxes delimit steps performed inside DIYA pipeline. Program names are in red font. See the text for details about each program. Abbreviations: FS, frameshifts.
(PDF)

Figure S4 Phylogenetic relationships of *Bartonella* showing the support for different placements of *Bartonella australis.* The numbered branches are the branches tested for the placement of *Bartonella australis*. For each single-gene tree, the placement of *B. australis* that gave the lowest log-likelihood among all five placements tested was determined. The table below the tree gives, for each possible placement, the number of single-gene trees for which the log-likelihood was the lowest, for both codon and amino-acid alignments.
(PDF)

Figure S5 Phylogenetic relationships of *Bartonella* inferred from different datasets. The phylogenies were inferred by maximum likelihood with 100 bootstraps, using different subsets of the concatenated alignment with different sequence divergence levels. The bootstrap support is given for representative phylogenies: A, nucleotide full concatenation; B, Amino-acid full concatenation; C, Amino-acid full concatenation, 10–15% divergence level. Unless otherwise shown, all nodes are supported by 100 bootstraps.
(PDF)

Figure S6 Phylogenetic relationships of *Bartonella* showing the support for different placements of the root. The numbered branches are the branches tested for the placement of the root in the ingroup. For each single-gene tree, the placement of the root that gave the lowest likelihood among all seven placements tested was determined. The table above the tree gives, for each possible placement, the number of single-gene trees for which the likelihood was the lowest, for both codon and amino-acid alignments.
(PDF)

Figure S7 Phylogeny of the *Bartonella* clade after removing the 10% most discordant genes. Maximum-likelihood phylogeny based on the concatenation of protein sequences of the least discordant genes. Bootstrap support is shown above the branches, and the scale is indicated below the tree. Abbreviations of *Bartonella* species are as in Table 1, except for BTa239 (*Bartonella tamiae* Th239). Abbreviations of outgroup species as in Figure 2.
(PDF)

Figure S8 Effect of removing discordant genes on the support values of critical splits. The x-axis represents the percentage of genes removed, removing the most discordant first. The y-axis represents the bootstrap support for four critical splits in separate maximum-likelihood phylogenies. The splits considered here are: *B. australis* NH1 clustering with the outgroup including *B. tamiae* (“BAnh1 first”, red), or with group B species (“BAnh1 with B”, green); *B. bacilliformis* clustering with the outgroup including *B. tamiae* (“BB first”, purple), or with group A species (“BB with A”, orange). For more details about removing discordant genes, see Material and Methods.
(PDF)

Figure S9 Genomic locations of the gene clusters for type IV secretion systems. Everything as in Figure 4, except that only the type IV secretion systems and rRNA operons are represented, each with a different color (see legend in the figure).
(PDF)

Figure S10 Phylogeny inferred from genes encoding type IV secretion system components. The phylogeny was inferred by maximum likelihood methods using amino acid alignments of a representative set of *virB4* homologs [41], in addition to the *Bartonella* genes. Some branches have been collapsed for visibility reasons. Color coding: red, *virB*; blue, *vbh*; green, *trw*; orange, *tra*. Support values above 90% from 100 bootstrap replicates are shown on the branches.
(PDF)

Figure S11 Phylogeny inferred from genes encoding the flagellum and type III secretion system components. The phylogeny was inferred by the maximum likelihood method using amino acid alignments of a representative set of the *flhA* and *sctV* homologs [81], in addition to the *Bartonella* and the outgroup species. Support values above 90% from 100 bootstrap replicates are shown on the branches. Color coding: blue, flagellar genes; green, type III secretion system genes; red, *Bartonella*; Vertical black bars indicate Alphaproteobacteria. Abbreviations: AP, Alphaproteobacteria.
(PDF)

Figure S12 Phylogeny inferred from the duplicated *impA* genes. The phylogeny was inferred by Bayesian methods using amino acid alignments of all identified homologs of this protein family. Color coding: blue, *B. bacilliformis* (BB); red, *B. australis* (BAnh1); dark red/dark green, *L. interrrogans* (Lin); yellow/light green, *L. borgpetersenii* (Lbo), black *Helicobacter hepaticus* (Hhe) and *H. mustelae* (Hmu), respectively. Ratio of support values from 100 maximum likelihood bootstrap replicates and Bayesian posterior probabilities are depicted above and below the branches, respectively.
(PDF)

Figure S13 *In vitro* transduction of a kanamycin resistance gene from *B. henselae* strain Marseille31 to strain Houston-1. The left panel shows the result of incubating phages extracted from the kanamycin-resistant *B. henselae* strain Marseille31 with kanamycin-sensitive strain Houston-1. The right panel shows a negative control in which no recipient kanamycin-sensitive bacteria were added.
(PDF)

Table S1 Assembly statistics for all *Bartonella* strains sequenced in this study. Abbreviations of *Bartonella* species names are as in Table 1.
(PDF)

Table S2 Genome statistics for all *Bartonella* strains analyzed in this study. Abbreviations of *Bartonella* species names are as in Table 1.
(PDF)

Table S3 Pair-wise nucleotide substitution frequencies at nonsynonymous (Ka) (lower triangle) and synonymous (Ks) (upper triangle) sites for all *Bartonella* strains and outgroup species analyzed in this study. Values of -1 indicate saturation. Abbreviations of *Bartonella* species names are as in Table 1.
(PDF)

Table S4 Genes and gene families inferred to have been acquired by the *Bartonella* last common ancestor.
(PDF)

Table S5 Averaged nucleotide substitution frequencies at nonsynonymous (Ka) and synonymous (Ks) sites for genes encoding the GTA and the ROR as well as for flanking sets of core genes.
(PDF)

Table S6 Average distances from the ROR origin to BaGTA, the first rRNA operon (rrs-1) and the *trw* operon, and size of the Secretion System Cassette (from *rrs*-1 to *trw*) in kb, for complete genomes and *B. bovis* 91-4, which consists of a single scaffold.
(PDF)
