# Supplementary figures and images for: A Gene Transfer Agent and a Dynamic Repertoire of Secretion Systems Hold the Keys to the Explosive Radiation of the Emerging Pathogen Bartonella
Source: PLoS Genet. 2013 Mar 28;9(3):e1003393. doi: 10.1371/journal.pgen.1003393 (PMC3610622; doi:10.1371/journal.pgen.1003393)

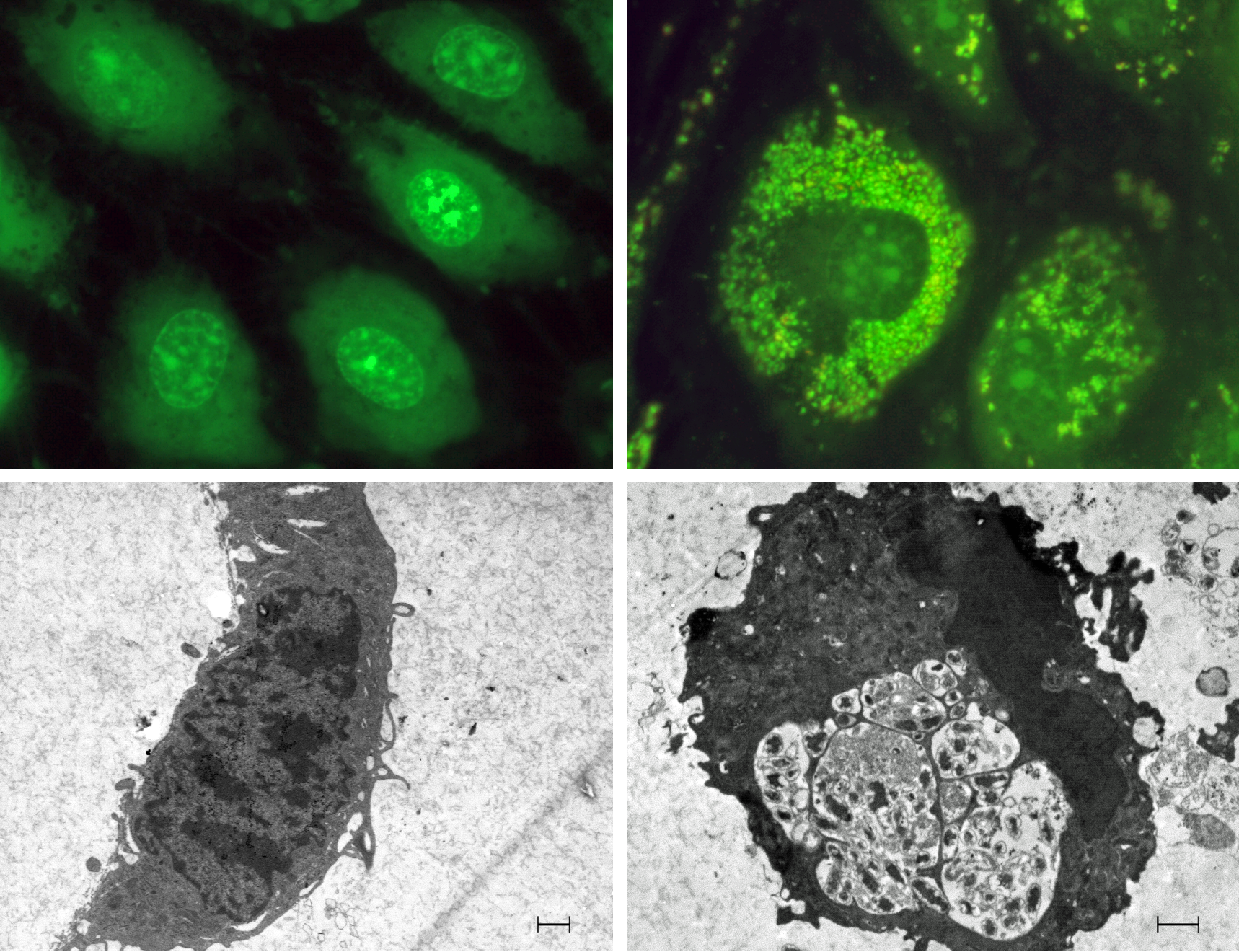

Supplement: Figure S1 — Infection of endothelial cells by Bartonella bovis m02. Acridine orange staining (upper panels) and transmission electron microscopy (lower panels) of bovine aortic endothelial cells both during uninfected conditions (left panels) and at 72 hours post infection (right panels) with B. bovis strain m02. The intracellular bacterial aggregates are visible as green dots around the nucleus (upper right panel) and as dark spots inside light grey vacuoles (lower right panel). The scale in each electron micrograph is indicated by a black bar (1 µm). (TIFF) [file pgen.1003393.s001.tiff]

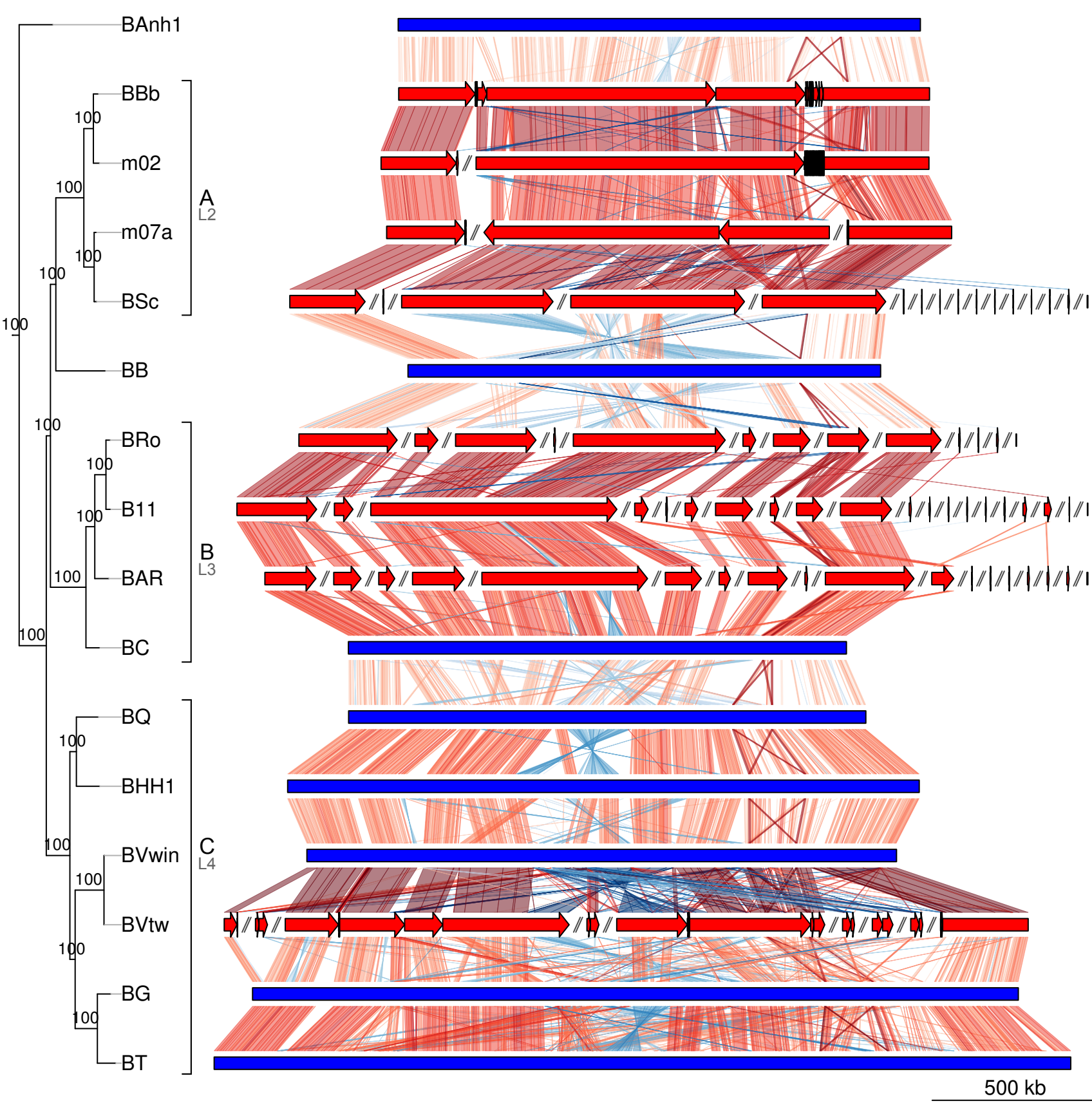

Supplement: Figure S2 — Map of contigs and scaffolds in the 16 Bartonella genomes. Complete and closed genomes are represented as blue lines, draft genomes as red arrows. Each arrow represents a contig. Scaffolds are separated by “//”. In B. bovis 91-4 and m02, B. schoenbuchensis m07a and B. vinsonii berkhoffii Tweed, the contig that spans the origin of replication has been broken up, resulting in a linear representation of the rightmost contig in the genome. Contigs that have not been placed within the scaffolds and that do not map to the genome of comparison are added at the right of the linear representation of the genome. The tree topology and the genome scale bar are as in Figure 4. The pairwise comparisons are as in Figure 4, except that direct hits are represented in red and complementary hits in blue. (PDF) [file pgen.1003393.s002.pdf]

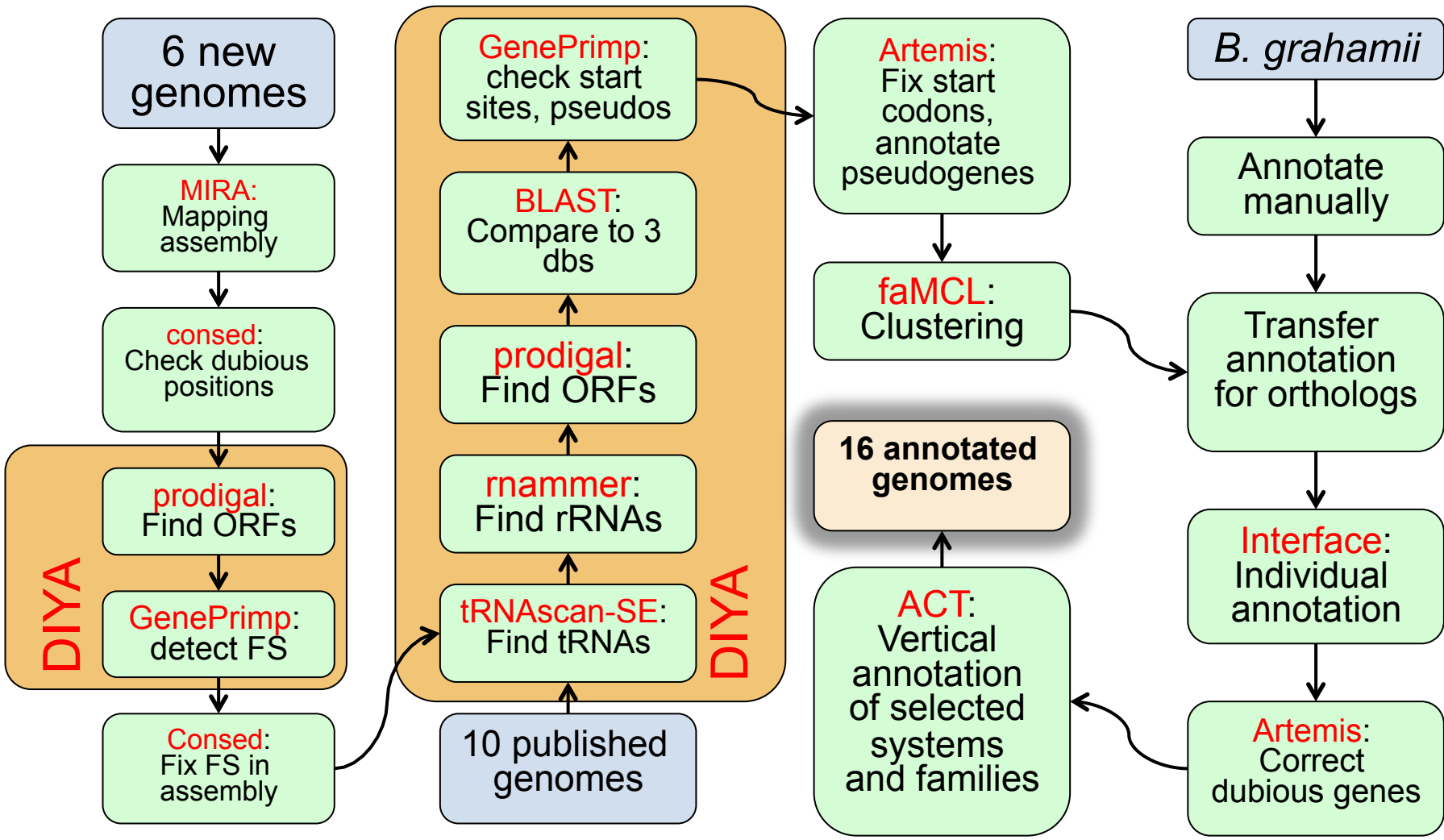

Supplement: Figure S3 — Overview of the annotation process. Blue background in boxes represent input data and green color a step in the pipeline. Boxes surrounded by larger orange boxes delimit steps performed inside DIYA pipeline. Program names are in red font. See the text for details about each program. Abbreviations: FS, frameshifts. (PDF) [file pgen.1003393.s003.pdf]

**Tree tested:**

|                | 1           | 2    | 3    | 4   | 5    |
|----------------|-------------|------|------|-----|------|
| baseml (codon) | <b>298</b>  | 22   | 40   | 1   | 67   |
| % of total     | <b>69.6</b> | 5.1  | 9.3  | 0.2 | 15.7 |
| codeml (AA)    | <b>153</b>  | 65   | 61   | 8   | 141  |
| % of total     | <b>35.7</b> | 15.2 | 14.3 | 1.9 | 32.9 |

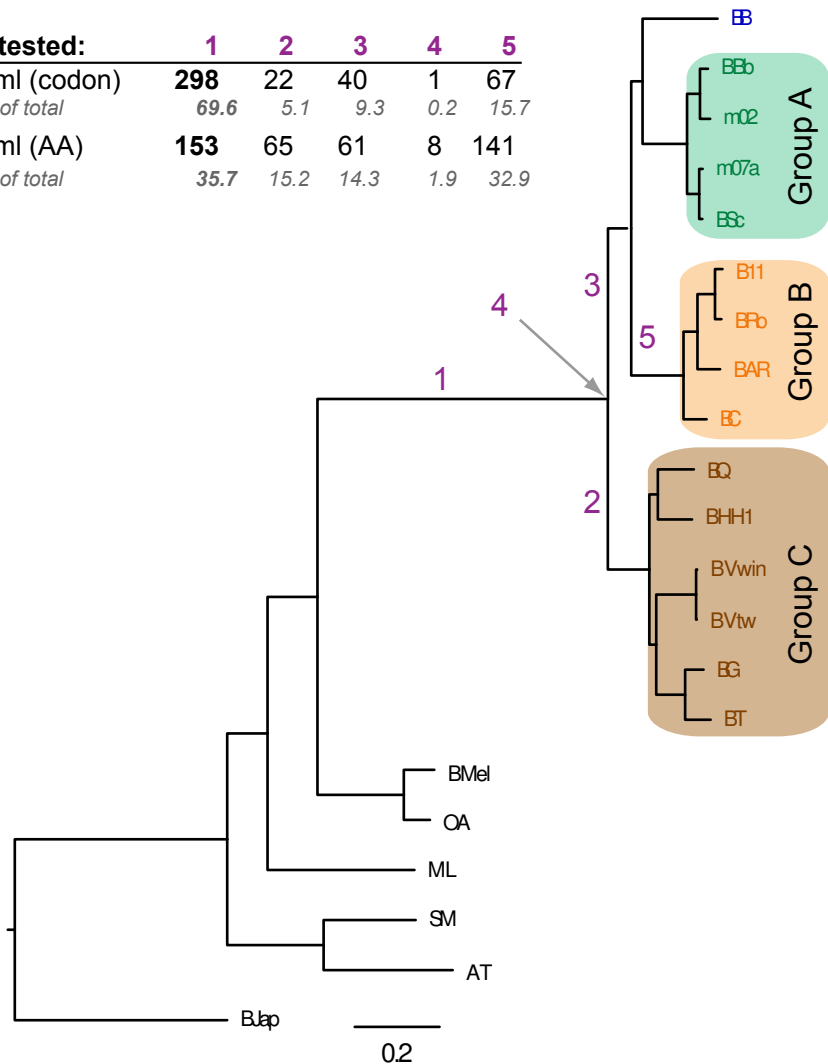

Supplement: Figure S4 — Phylogenetic relationships of Bartonella showing the support for different placements of Bartonella australis. The numbered branches are the branches tested for the placement of Bartonella australis. For each single-gene tree, the placement of B. australis that gave the lowest log-likelihood among all five placements tested was determined. The table below the tree gives, for each possible placement, the number of single-gene trees for which the log-likelihood was the lowest, for both codon and amino-acid alignments. (PDF) [file pgen.1003393.s004.pdf]

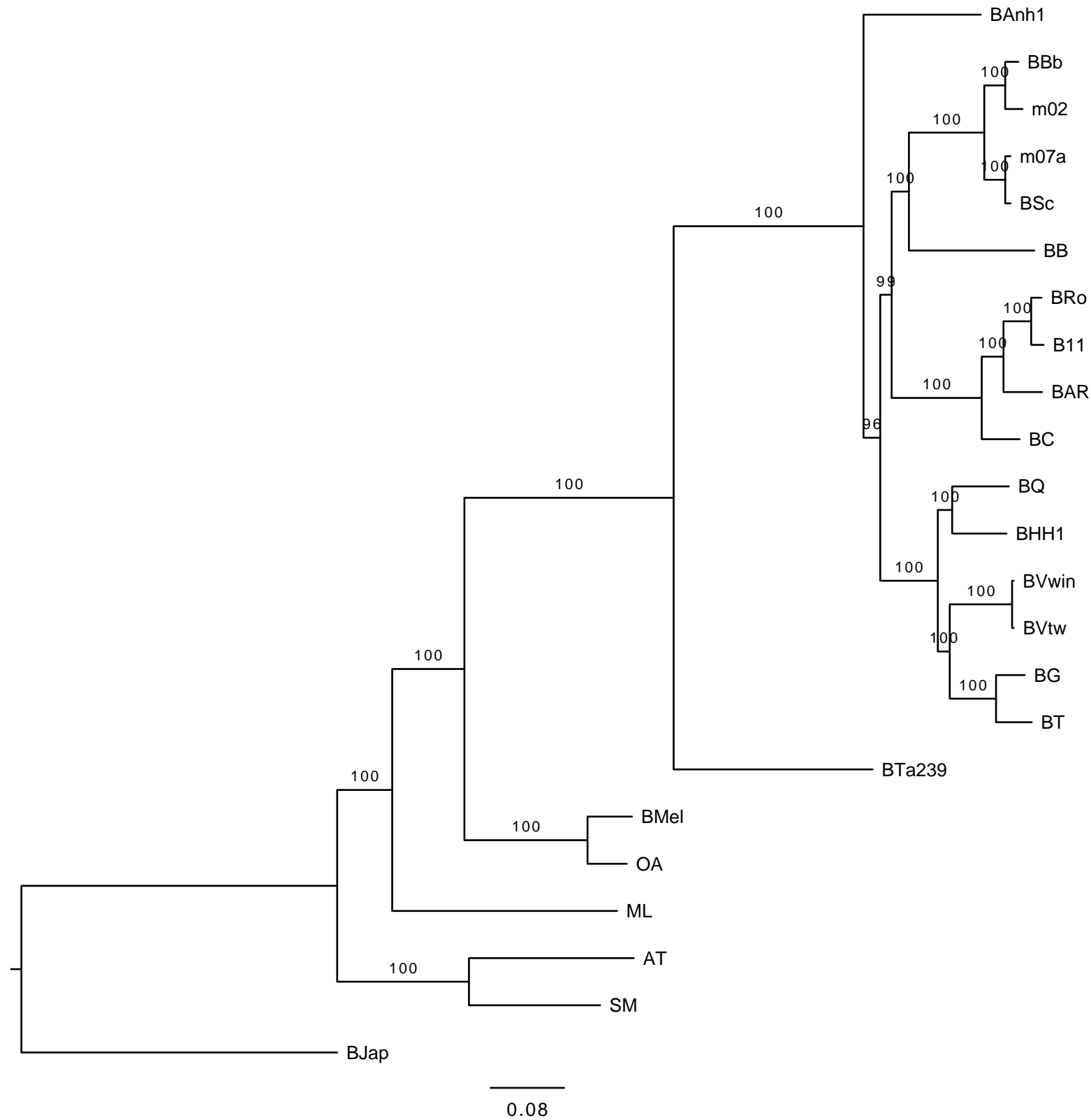

Supplement: Figure S7 — Phylogeny of the Bartonella clade after removing the 10% most discordant genes. Maximum-likelihood phylogeny based on the concatenation of protein sequences of the least discordant genes. Bootstrap support is shown above the branches, and the scale is indicated below the tree. Abbreviations of Bartonella species are as in Table 1, except for BTa239 (Bartonella tamiae Th239). Abbreviations of outgroup species as in Figure 2. (PDF) [file pgen.1003393.s007.pdf]

## Discordance filter

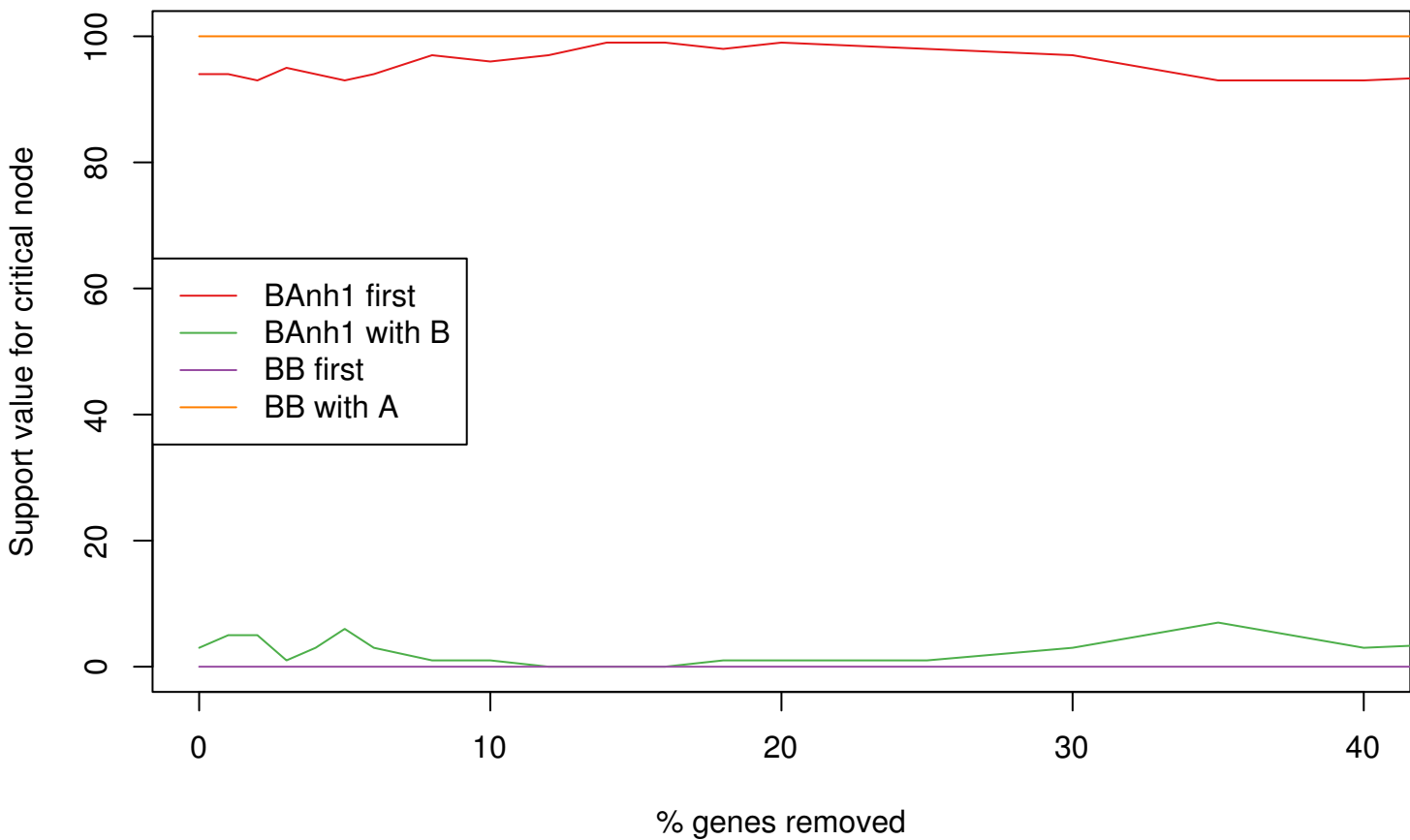

Supplement: Figure S8 — Effect of removing discordant genes on the support values of critical splits. The x-axis represents the percentage of genes removed, removing the most discordant first. The y-axis represents the bootstrap support for four critical splits in separate maximum-likelihood phylogenies. The splits considered here are: B. australis NH1 clustering with the outgroup including B. tamiae (“BAnh1 first”, red), or with group B species (“BAnh1 with B”, green); B. bacilliformis clustering with the outgroup including B. tamiae (“BB first”, purple), or with group A species (“BB with A”, orange). For more details about removing discordant genes, see Material and Methods. (PDF) [file pgen.1003393.s008.pdf]

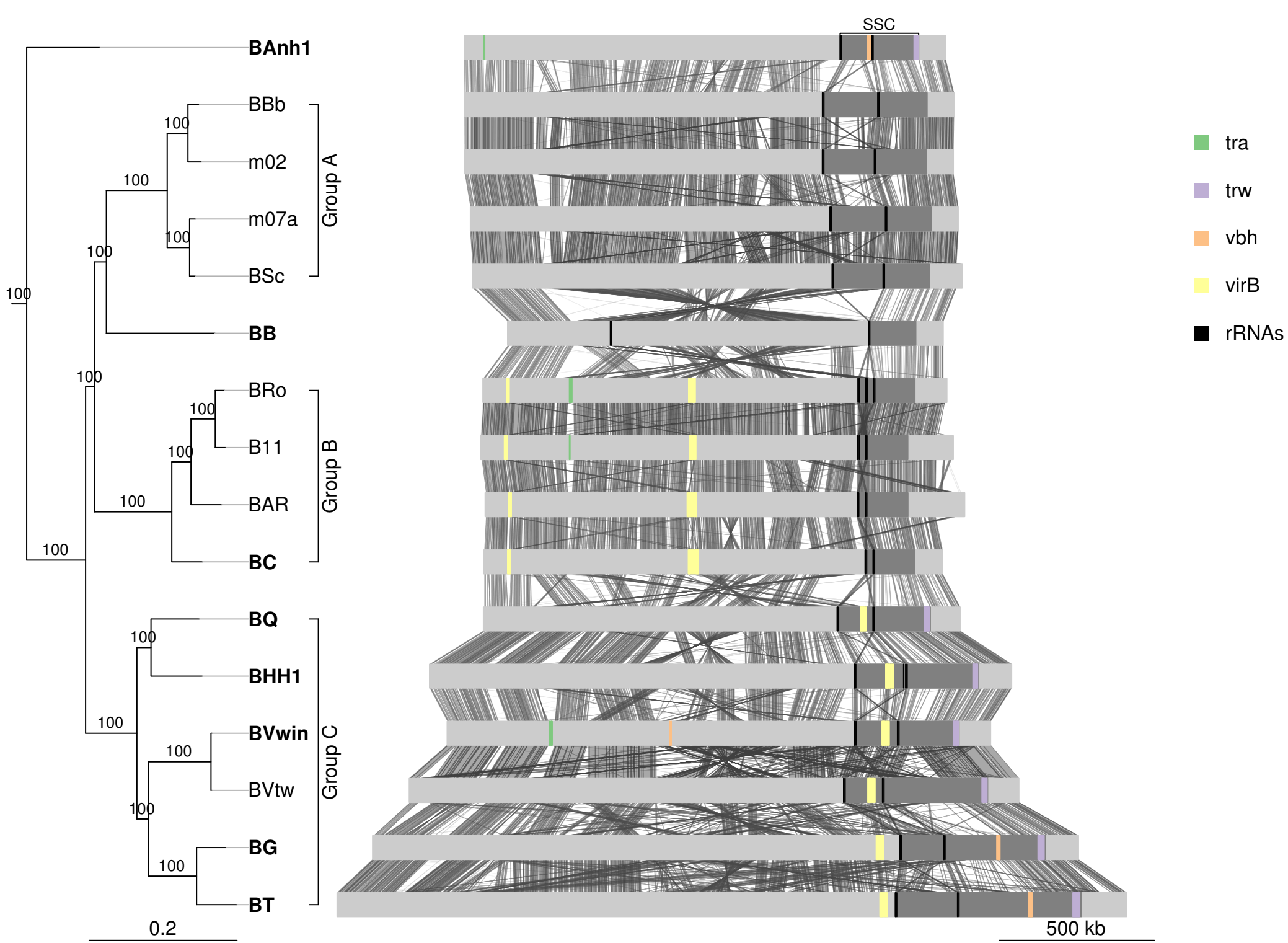

Supplement: Figure S9 — Genomic locations of the gene clusters for type IV secretion systems. Everything as in Figure 4, except that only the type IV secretion systems and rRNA operons are represented, each with a different color (see legend in the figure). (PDF) [file pgen.1003393.s009.pdf]

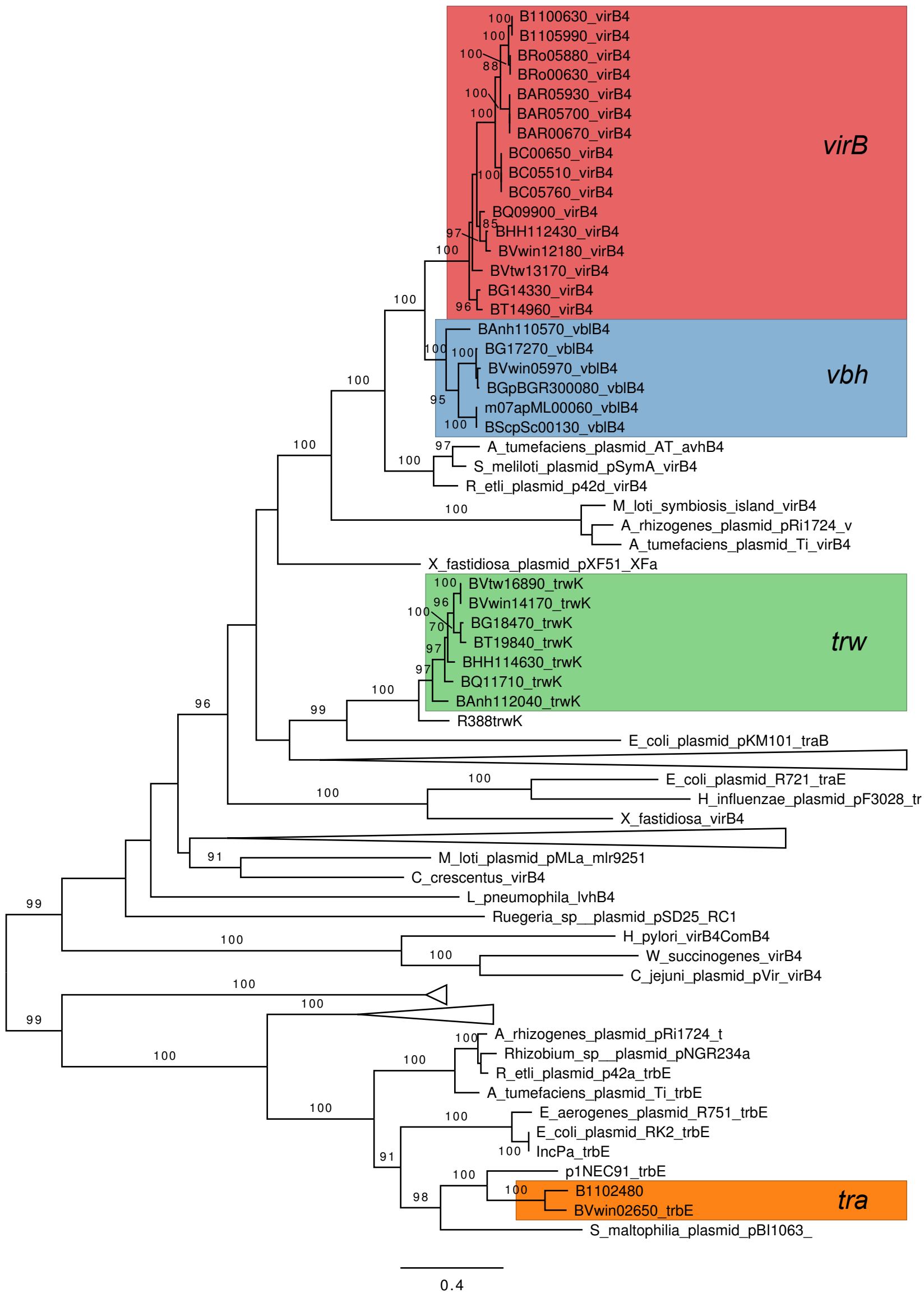

Supplement: Figure S10 — Phylogeny inferred from genes encoding type IV secretion system components. The phylogeny was inferred by maximum likelihood methods using amino acid alignments of a representative set of virB4 homologs [41], in addition to the Bartonella genes. Some branches have been collapsed for visibility reasons. Color coding: red, virB; blue, vbh; green, trw; orange, tra. Support values above 90% from 100 bootstrap replicates are shown on the branches. (PDF) [file pgen.1003393.s010.pdf]

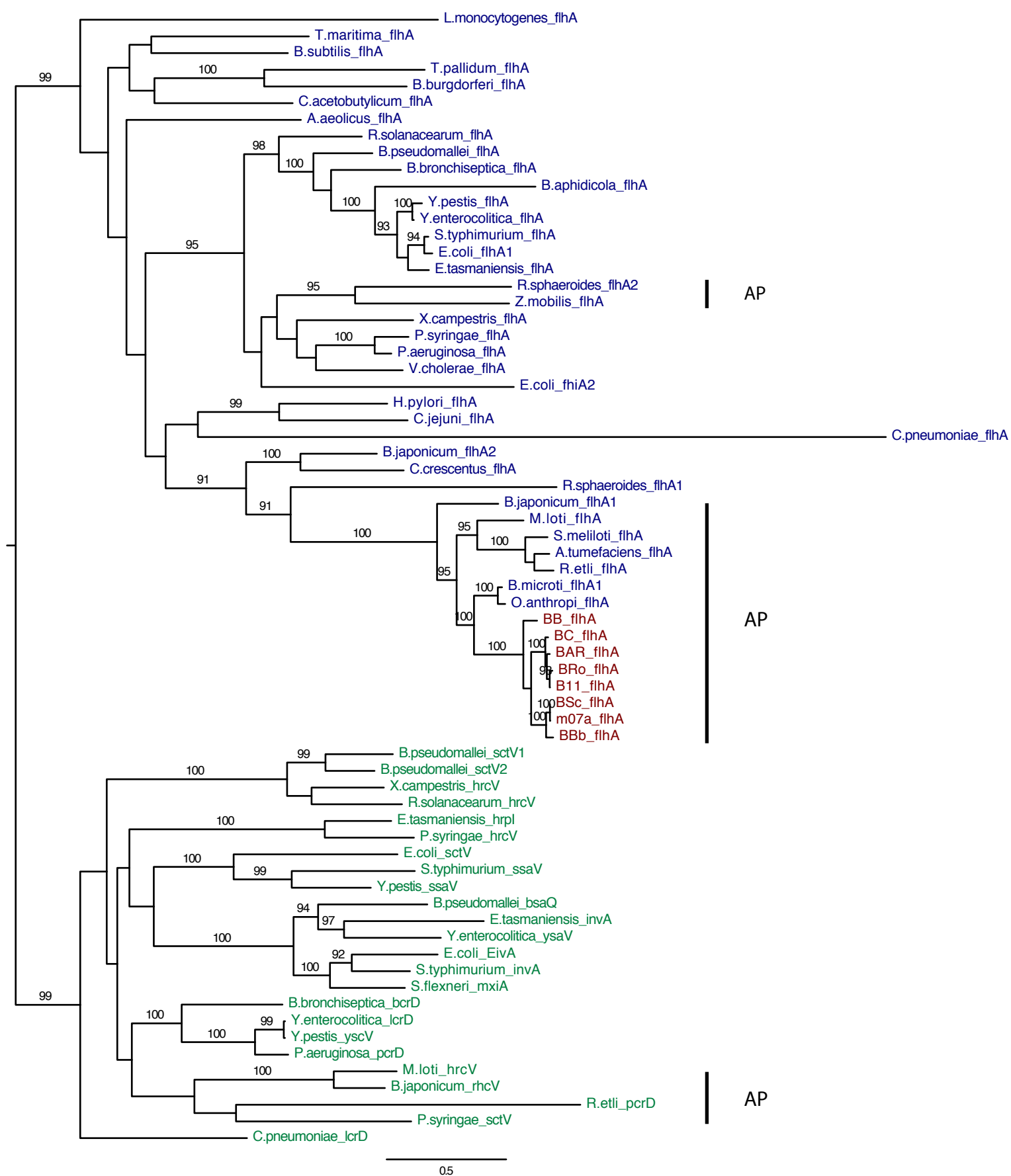

Supplement: Figure S11 — Phylogeny inferred from genes encoding the flagellum and type III secretion system components. The phylogeny was inferred by the maximum likelihood method using amino acid alignments of a representative set of the flhA and sctV homologs [81], in addition to the Bartonella and the outgroup species. Support values above 90% from 100 bootstrap replicates are shown on the branches. Color coding: blue, flagellar genes; green, type III secretion system genes; red, Bartonella; Vertical black bars indicate Alphaproteobacteria. Abbreviations: AP, Alphaproteobacteria. (PDF) [file pgen.1003393.s011.pdf]

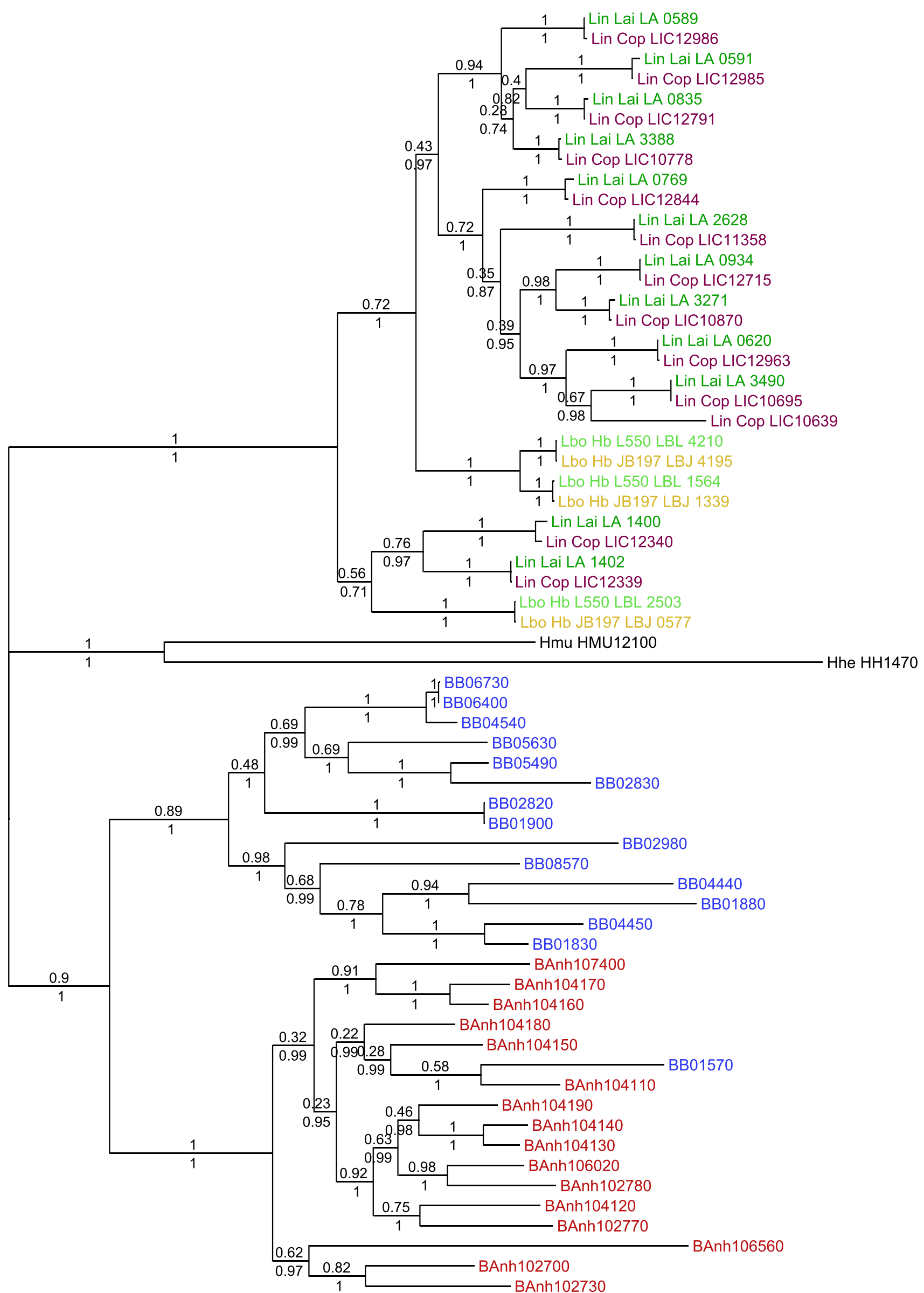

Supplement: Figure S12 — Phylogeny inferred from the duplicated impA genes. The phylogeny was inferred by Bayesian methods using amino acid alignments of all identified homologs of this protein family. Color coding: blue, B. bacilliformis (BB); red, B. australis (BAnh1); dark red/dark green, L. interrrogans (Lin); yellow/light green, L. borgpetersenii (Lbo), black Helicobacter hepaticus (Hhe) and H. mustelae (Hmu), respectively. Ratio of support values from 100 maximum likelihood bootstrap replicates and Bayesian posterior probabilities are depicted above and below the branches, respectively. (PDF) [file pgen.1003393.s012.pdf]

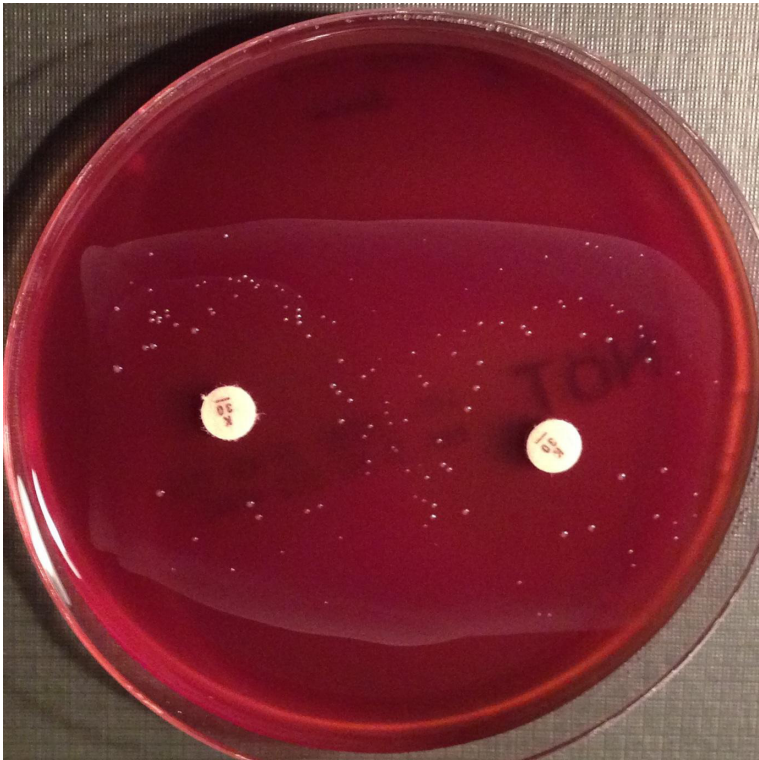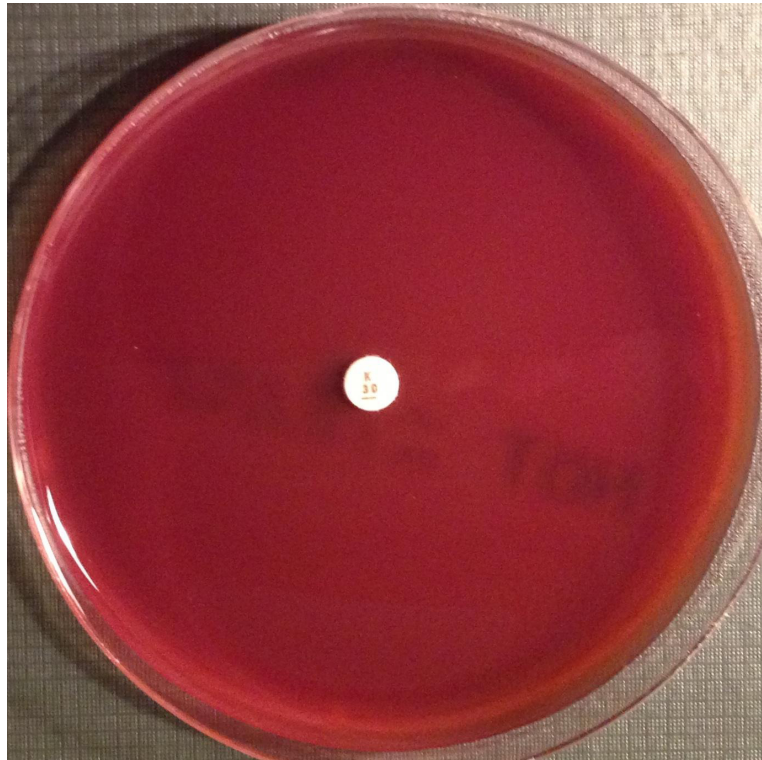

Supplement: Figure S13 — In vitro transduction of a kanamycin resistance gene from B. henselae strain Marseille31 to strain Houston-1. The left panel shows the result of incubating phages extracted from the kanamycin-resistant B. henselae strain Marseille31 with kanamycin-sensitive strain Houston-1. The right panel shows a negative control in which no recipient kanamycin-sensitive bacteria were added. (PDF) [file pgen.1003393.s013.pdf]
