# Supplementary material for: A Gene Transfer Agent and a Dynamic Repertoire of Secretion Systems Hold the Keys to the Explosive Radiation of the Emerging Pathogen Bartonella
Source: PLoS Genet. 2013 Mar 28;9(3):e1003393. doi: 10.1371/journal.pgen.1003393 (PMC3610622; doi:10.1371/journal.pgen.1003393)
Supplement: Figure S5 — Phylogenetic relationships of Bartonella inferred from different datasets. The phylogenies were inferred by maximum likelihood with 100 bootstraps, using different subsets of the concatenated alignment with different sequence divergence levels. The bootstrap support is given for representative phylogenies: A, nucleotide full concatenation; B, Amino-acid full concatenation; C, Amino-acid full concatenation, 10–15% divergence level. Unless otherwise shown, all nodes are supported by 100 bootstraps. (PDF) [file pgen.1003393.s005.pdf]

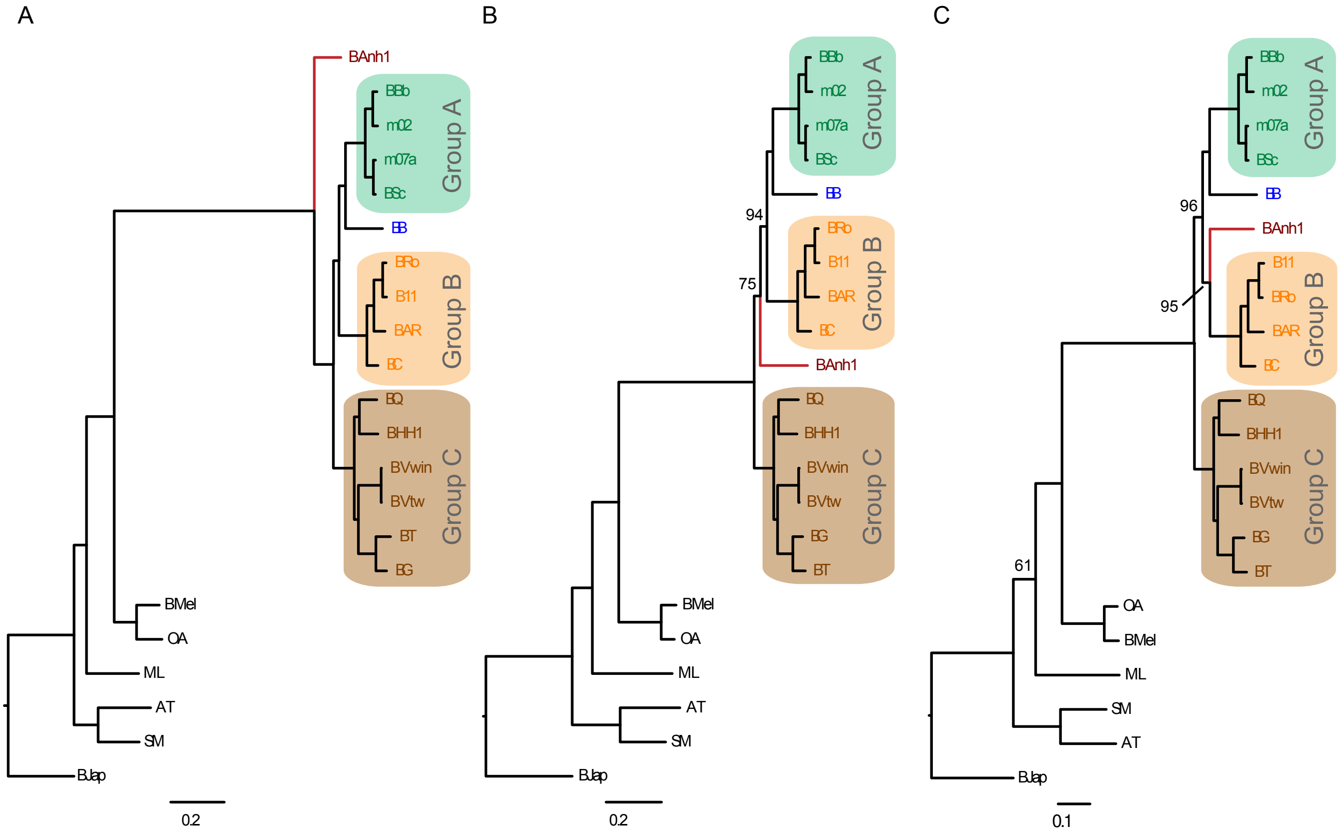

Datasets	Best tree picked by RAxML for each of the alignments		
Nucleotide	Full concatenation<br>All divergence level concatenations	none	none
Amino acid	15-20% divergence level<br>20-25% divergence level	Full concatenation	5-10% divergence level<br>10-15% divergence level
Nucleotide without 3rd codon positon	15-20% divergence level<br>20-25% divergence level	Full concatenation<br>10-15% divergence level	5-10% divergence level
