# Supplementary material for: A Gene Transfer Agent and a Dynamic Repertoire of Secretion Systems Hold the Keys to the Explosive Radiation of the Emerging Pathogen Bartonella
Source: PLoS Genet. 2013 Mar 28;9(3):e1003393. doi: 10.1371/journal.pgen.1003393 (PMC3610622; doi:10.1371/journal.pgen.1003393)
Supplement: Figure S6 — Phylogenetic relationships of Bartonella showing the support for different placements of the root. The numbered branches are the branches tested for the placement of the root in the ingroup. For each single-gene tree, the placement of the root that gave the lowest likelihood among all seven placements tested was determined. The table above the tree gives, for each possible placement, the number of single-gene trees for which the likelihood was the lowest, for both codon and amino-acid alignments. (PDF) [file pgen.1003393.s006.pdf]

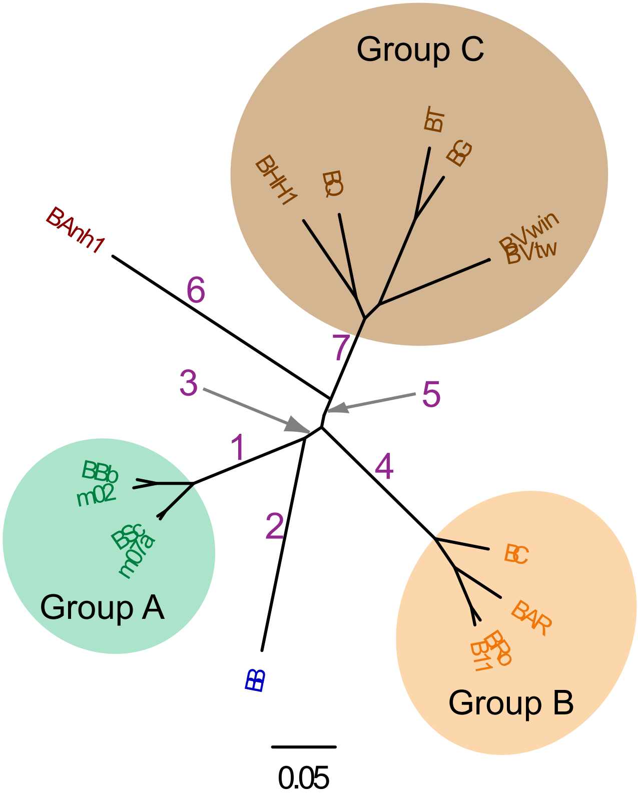

**Tree tested:**	**1**	**2**	**3**	**4**	**5**	**6**	**7**
baseml (codon)	32	71	3	22	1	**259**	40
% of total	*7.5*	*16.6*	*0.7*	*5.1*	*0.2*	***60.5***	*9.3*
codeml (AA)	43	93	8	52	14	**127**	91
% of total	*10*	*21.7*	*1.9*	*12.1*	*3.3*	***29.7***	*21.3*
