# Supplementary material for: A Gene Transfer Agent and a Dynamic Repertoire of Secretion Systems Hold the Keys to the Explosive Radiation of the Emerging Pathogen Bartonella
Source: PLoS Genet. 2013 Mar 28;9(3):e1003393. doi: 10.1371/journal.pgen.1003393 (PMC3610622; doi:10.1371/journal.pgen.1003393)
Supplement: Table S1 — Assembly statistics for all Bartonella strains sequenced in this study. Abbreviations of Bartonella species names are as in Table 1. (PDF) [file pgen.1003393.s014.pdf]

	BAnh1	BBb	m02	m07a	BVwin	BVtw
Genome shotgun sequence data						
Number of reads (Sanger)	0	0	16,000	0	20,000	0
Number of bases (Sanger)	0	0	10 Mb	0	16 Mb	0
Read coverage (Sanger)	-	-	5X	-	9X	-
Number of reads (454)	370,000	930,000	280,000	320,000	160,000	380'000
Number of bases (454)	113 Mb	310 Mb	15 Mb	106 Mb	38 Mb	125 Mb
Instrument (454)	Titanium	Titanium	GS20	Titanium	FLX	Titanium
Read coverage (454)	60X	120X	10X	60X	20X	60X
Number of reads (Illumina SIPE)	3.7 M	12 M	3.4 M	2.8 M	-	3.1 M
Number of bases (Illumina SIPE)	140 Mb	655 Mb	131 Mb	105 Mb	-	118 Mb
Number of reads (Illumina LIPE)	-	4.1 M	-	-	-	-
Number of bases (Illumina LIPE)	-	158 Mb	-	-	-	-
Read coverage (Illumina)	100X	480X	80X	60X	-	60X
Paired-end library, 20 kb inserts						
Number of read-pairs (454)	6500	0	0	4400	0	7500
PCR:s						
Number of PCR:s tried	77	0	353	108	247	100
Number of sequenced PCR products	11	0	29	15	26	24
Fosmids						
Number of sequenced fosmid ends	200	400	0	600	0	200
Number of sequenced fosmids	6	10	5	18	0	12
Estimated genome size (PFGE)	1.7 Mb	1.7 Mb	1.7 Mb	1.8 Mb	2.0 Mb	
Assembly						
Assembly programs [A]	M,P	M,P	G,P	M,P	G,P	M,P
Number of scaffolds	1	2	1	2	1	1
Number of contigs (>1 kb)	1	17	3*	8	1	25
Number of reads	370,000	930,000	293,000	320,000	180,000	380,000
Number of bases	113 Mb	310 Mb	25 Mb	106 Mb	54 Mb	125 Mb
Read coverage (without Illumina)	60X	120X	15X	60X	30X	60X
Total size	1.6 Mb	1.5 Mb	1.6 Mb	1.7 Mb	1.8 Mb	2.0 Mb
Estimated genome coverage (%)**	100%	95%	95%	95%	100%	95%
Status	Complete	Late draft	Late draft	Late draft	Complete	Draft

* Two large repeat regions excluded from this number
** Correctly assembled
[A] G = GS Assembler, M = MIRA, P = Phrap
