# Supplementary material for: A Gene Transfer Agent and a Dynamic Repertoire of Secretion Systems Hold the Keys to the Explosive Radiation of the Emerging Pathogen Bartonella
Source: PLoS Genet. 2013 Mar 28;9(3):e1003393. doi: 10.1371/journal.pgen.1003393 (PMC3610622; doi:10.1371/journal.pgen.1003393)
Supplement: Table S2 — Genome statistics for all Bartonella strains analyzed in this study. Abbreviations of Bartonella species names are as in Table 1. (PDF) [file pgen.1003393.s015.pdf]

ID	Size (Mb)	Coding frac.	GC cont.	CDS:s	pseudo	tRNA:s	rRNA:s*	phage
BAnh1	1.60	0.8	0.42	1265	20	42	2	118
BB	1.45	0.81	0.38	1157	36	44	2	38
BBb	1.62	0.81	0.37	1298	28	43	2	142
m02	1.62	0.8	0.37	1296	44	42	2	159
m07a	1.68	0.82	0.38	1421	16	42	2	181
m07a [C]	1.62	0.82	0.38	1335	12	42	2	181
m07apML [P]	0.06	0.74	0.35	83	4	0	0	0
m07apMS [P]	0.002	0.6	0.4	3	0	0	0	0
BSc	1.67	0.8	0.38	1410	26	42	2	236
BSc [C]	1.62	0.81	0.38	1350	23	42	2	236
BScpSc [P]	0.04	0.77	0.35	60	3	0	0	0
BRo	1.54	0.79	0.36	1224	47	44	3	47
B11	1.57	0.8	0.36	1285	42	41	2	90
BAR	1.59	0.8	0.36	1284	53	41	2	121
BC	1.52	0.81	0.36	1193	21	41	2	21
BHH1	1.93	0.72	0.38	1430	124	43	2	188
BQ	1.58	0.73	0.39	1135	131	42	2	31
BVwin	1.80	0.77	0.39	1436	71	42	2	193
BVtw	2.02	0.75	0.4	1650	117	41	2	441
BG	2.37	0.73	0.38	1749	240	41	2	329
BG [C]	2.34	0.73	0.38	1718	240	41	2	329
BGpBGR3 [P]	0.03	0.83	0.36	31	0	0	0	0
BT	2.64	0.73	0.39	2047	102	42	2	615
BT [C]	2.62	0.73	0.39	2029	102	42	2	615
BTpBtr [P]	0.02	0.71	0.35	18	0	0	0	0

* Number of complete rRNA operons
[C] Chromosome
[P] Plasmid
