# Supplementary material for: A Gene Transfer Agent and a Dynamic Repertoire of Secretion Systems Hold the Keys to the Explosive Radiation of the Emerging Pathogen Bartonella
Source: PLoS Genet. 2013 Mar 28;9(3):e1003393. doi: 10.1371/journal.pgen.1003393 (PMC3610622; doi:10.1371/journal.pgen.1003393)
Supplement: Table S3 — Pair-wise nucleotide substitution frequencies at nonsynonymous (Ka) (lower triangle) and synonymous (Ks) (upper triangle) sites for all Bartonella strains and outgroup species analyzed in this study. Values of -1 indicate saturation. Abbreviations of Bartonella species names are as in Table 1. (PDF) [file pgen.1003393.s016.pdf]

	BAnh1	BBb	m02	m07a	BSc	BB	B11	BRo	BAR	BC	BQ	BHH1	BVwin	BWtw	BG	BT	BMel	OA	ML	SM	AT	BJap	Ks
BAnh1		0.90	0.90	0.90	0.90	0.97	0.99	0.99	0.97	0.94	0.95	0.98	1.01	1.01	1.05	1.09	-1	-1	-1	-1	-1	-1	BAnh1
BBb	0.11		0.09	0.25	0.25	0.68	0.70	0.70	0.69	0.66	0.70	0.72	0.75	0.75	0.77	0.81	-1	-1	-1	-1	-1	-1	BBb
m02	0.11	0.01		0.25	0.25	0.68	0.70	0.70	0.69	0.66	0.70	0.72	0.75	0.75	0.77	0.81	-1	-1	-1	-1	-1	-1	m02
m07a	0.10	0.03	0.03		0.05	0.68	0.70	0.70	0.70	0.66	0.70	0.72	0.75	0.75	0.77	0.81	-1	-1	-1	-1	-1	-1	m07a
BSc	0.10	0.03	0.03	0.00		0.67	0.70	0.70	0.69	0.66	0.70	0.72	0.74	0.74	0.77	0.80	-1	-1	-1	-1	-1	-1	BSc
BB	0.12	0.10	0.10	0.10	0.10		0.75	0.75	0.75	0.72	0.76	0.77	0.80	0.80	0.82	0.85	-1	-1	-1	-1	-1	-1	BB
B11	0.12	0.11	0.11	0.11	0.11	0.12		0.08	0.24	0.31	0.78	0.78	0.80	0.80	0.81	0.84	-1	-1	-1	-1	-1	-1	B11
BRo	0.12	0.11	0.11	0.11	0.11	0.12	0.01		0.24	0.30	0.78	0.78	0.80	0.80	0.81	0.84	-1	-1	-1	-1	-1	-1	BRo
BAR	0.12	0.11	0.11	0.11	0.11	0.12	0.04	0.03		0.29	0.77	0.78	0.79	0.79	0.81	0.85	-1	-1	-1	-1	-1	-1	BAR
BC	0.11	0.10	0.10	0.10	0.10	0.11	0.04	0.04	0.04		0.73	0.74	0.76	0.76	0.78	0.82	-1	-1	-1	-1	-1	-1	BC
BQ	0.11	0.10	0.10	0.10	0.10	0.11	0.11	0.11	0.11	0.11		0.38	0.51	0.51	0.55	0.60	-1	-1	-1	-1	-1	-1	BQ
BHH1	0.11	0.10	0.11	0.10	0.10	0.11	0.11	0.11	0.11	0.11	0.05		0.51	0.51	0.56	0.60	-1	-1	-1	-1	-1	-1	BHH1
BVwin	0.11	0.11	0.11	0.10	0.10	0.11	0.12	0.11	0.12	0.11	0.06	0.06		0.01	0.56	0.60	-1	-1	-1	-1	-1	-1	BVwin
BWtw	0.11	0.11	0.11	0.10	0.10	0.11	0.12	0.11	0.12	0.11	0.06	0.06	0.00		0.56	0.60	-1	-1	-1	-1	-1	-1	BWtw
BG	0.11	0.11	0.11	0.11	0.11	0.12	0.12	0.12	0.12	0.11	0.07	0.06	0.06	0.06		0.30	-1	-1	-1	-1	-1	-1	BG
BT	0.11	0.11	0.11	0.11	0.11	0.12	0.12	0.12	0.12	0.12	0.07	0.07	0.07	0.07	0.03		-1	-1	-1	-1	-1	-1	BT
BMel	0.29	0.29	0.29	0.29	0.29	0.29	0.30	0.30	0.30	0.29	0.29	0.29	0.29	0.29	0.29	0.29		0.73	1.02	1.13	1.08	1.12	BMel
OA	0.29	0.29	0.29	0.29	0.29	0.29	0.30	0.30	0.30	0.29	0.29	0.29	0.29	0.29	0.29	0.29	0.05		1.10	1.21	1.15	1.21	OA
ML	0.33	0.33	0.33	0.33	0.33	0.33	0.34	0.34	0.34	0.33	0.33	0.33	0.32	0.32	0.33	0.33	0.22	0.22		0.84	0.95	0.75	ML
SM	0.34	0.35	0.35	0.34	0.34	0.35	0.35	0.35	0.35	0.35	0.34	0.34	0.34	0.34	0.34	0.34	0.24	0.24	0.25		0.95	0.85	SM
AT	0.35	0.35	0.35	0.35	0.35	0.35	0.36	0.36	0.36	0.35	0.35	0.35	0.35	0.35	0.35	0.35	0.26	0.25	0.26	0.16		0.99	AT
BJap	0.43	0.44	0.44	0.43	0.43	0.44	0.44	0.44	0.44	0.44	0.43	0.43	0.43	0.43	0.43	0.43	0.37	0.36	0.36	0.37	0.38		BJap
Ka	BAnh1	BBb	m02	m07a	BSc	BB	B11	BRo	BAR	BC	BQ	BHH1	BVwin	BWtw	BG	BT	BMel	OA	ML	SM	AT	BJap	
