# Supplementary material for: A Gene Transfer Agent and a Dynamic Repertoire of Secretion Systems Hold the Keys to the Explosive Radiation of the Emerging Pathogen Bartonella
Source: PLoS Genet. 2013 Mar 28;9(3):e1003393. doi: 10.1371/journal.pgen.1003393 (PMC3610622; doi:10.1371/journal.pgen.1003393)
Supplement: Table S4 — Genes and gene families inferred to have been acquired by the Bartonella last common ancestor. (PDF) [file pgen.1003393.s017.pdf]

**Product**	**Gene families**	**Genes**
phage-related protein	23	685
hypothetical protein	48	729
T4SS-related protein (trwL, trwJ, korA, korB, virB7/vblB7)	5	102
surface protein/Bartonella adhesin (badA)	1	20
pertactin family virulence factor/autotransporter (autoB)	1	14
hypothetical protein,nuclease (SNase-like)	1	19
sodium/dicarboxylate symporter	1	16
DNA-damage-inducible protein J	1	16
putative virulence determinant	1	16
thiamine-phosphate pyrophosphorylase ThiE	1	16
cell wall hydrolase SleB	1	16
cytochrome c-type biogenesis protein CycH	1	16
ATP-dependent helicase (fragment)	1	15
SH3-domain protein	1	11
YacA protein	1	10
PemK protein	1	9
PemI protein	1	8
LysM domain/BON superfamily protein	1	7
orotidine-5'-phosphate decarboxylase	1	8
putative permease of the major facilitator superfamily	1	4
