# Supplementary material for: A Gene Transfer Agent and a Dynamic Repertoire of Secretion Systems Hold the Keys to the Explosive Radiation of the Emerging Pathogen Bartonella
Source: PLoS Genet. 2013 Mar 28;9(3):e1003393. doi: 10.1371/journal.pgen.1003393 (PMC3610622; doi:10.1371/journal.pgen.1003393)
Supplement: Table S5 — Averaged nucleotide substitution frequencies at nonsynonymous (Ka) and synonymous (Ks) sites for genes encoding the GTA and the ROR as well as for flanking sets of core genes. (PDF) [file pgen.1003393.s018.pdf]

**Region**	**Number of genes**	**Ka**	**Ks**
Core, upstream BaGTA	9-12	0.100 +/- 0.14	0.57 +/- 0.40
BaGTA	11-13	0.098 +/- 0.11	0.54 +/- 0.37
Core, inter BaGTA-ROR	9-12	0.073 +/- 0.07	0.60 +/- 0.42
ROR	6-7	0.095 +/- 0.08	0.53 +/- 0.34
Core, downstream ROR	10	0.095 +/- 0.11	0.49 +/- 0.45
Genome-wide core genes	428	0.044 +/- 0.02	0.37 +/- 0.19
