# Supplementary material for: A Gene Transfer Agent and a Dynamic Repertoire of Secretion Systems Hold the Keys to the Explosive Radiation of the Emerging Pathogen Bartonella
Source: PLoS Genet. 2013 Mar 28;9(3):e1003393. doi: 10.1371/journal.pgen.1003393 (PMC3610622; doi:10.1371/journal.pgen.1003393)
Supplement: Table S6 — Average distances from the ROR origin to BaGTA, the first rRNA operon (rrs-1) and the trw operon, and size of the Secretion System Cassette (from rrs-1 to trw) in kb, for complete genomes and B. bovis 91-4, which consists of a single scaffold. (PDF) [file pgen.1003393.s019.pdf]

	ROR to BaGTA	ROR to rrs-1	ROR to trw	rrs-1 to trw
BAnh1	23	151	100	258
BBb	29	239	-	345
BB	35	(59) [a]	-	(155) [a]
BC	26	75	-	162
BQ	32	174	122	306
BHH1	49	248	152	409
BVwin	30	197	138	344
BG	91	262	205	481
BT	82	319	280	615
Median	***32***	***197***	***145***	***344***
Median w/o BC and BB [b]	*31*	*218*	*145*	*344.5*

[a] Due to the large inversion in *B. bacilliformis*, these distances have been estimated from the second break-point of the inversion, located just upstream from rrs-2, and is thus artificially smaller.

[b] Due to the major rearrangements in *B. bacilliformis* and in group B strains, a separate statistic is shown for groups A, C and BAnh1.
